# Graph-Based Internal Coordinate Analysis for Transition State Characterization

**DOI:** 10.1021/acs.jctc.5c02073

**Published:** 2026-02-21

**Authors:** Alister S. Goodfellow, Bao N. Nguyen

**Affiliations:** School of Chemistry, 4468 University of Leeds, Woodhouse Lane, Leeds LS2 9JT, UK

## Abstract

We present graphRC, a method for rapid transition state (TS) mode analysis using internal coordinates derived from molecular graphs. The imaginary mode of a TS describes the direction of atomic motion at the saddle point, providing a local approximation to the reaction coordinate, while Intrinsic Reaction Coordinate (IRC) and Quick Reaction Coordinate (QRC) calculations trace the full pathway to adjacent minima. In all cases, displacements are expressed in Cartesian coordinates and do not directly describe changes in bonding. By translating these into internal coordinate changes (bonds, angles, and dihedrals), graphRC provides chemical insight into the TS mode and reaction coordinate trajectories without prior knowledge of reactant and product structures. Molecular connectivity is determined using xyzgraph, a flexible graph builder validated across 4846 structures spanning 61 elements and 490 element-pair bond types, with close agreement to DFT-derived bonding. Initial validation on 16 diverse TS achieved 100% identification of bond changes, rotations, and inversions, with zero false positives compared to IRC and QRC connectivity. Across 395 TS covering organic, organometallic, and catalytic transformations, normal-mode analysis alone detects the primary bond change in every case, with high agreement to IRC-derived connectivity. This enables programmatic TS verification at a fraction of the cost of formal reaction coordinate calculations, complementing more rigorous methods with rapid, interpretable analysis.

## Graphical abstract

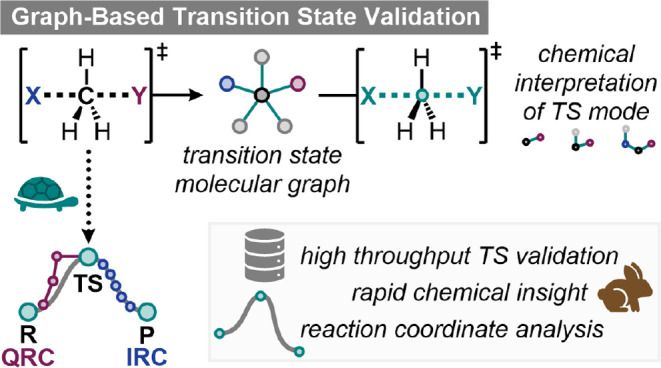

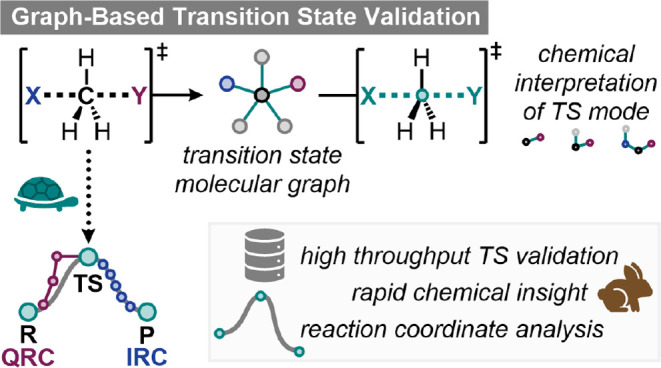

## Introduction

Transition state (TS) validation is central to mechanistic computational chemistry but remains a major bottleneck for high-throughput computational workflows. Formal verification is performed using Intrinsic Reaction Coordinate (IRC) calculations, which trace the minimum-energy pathway downhill from the transition state to adjacent minima (Figure 1A).^1–3^ While IRC calculations are the gold standard, they are computationally expensive, typically requiring reevaluation of the Hessian and very small displacement steps to accurately map out the Potential Energy Surface (PES). These calculations can be challenging to perform and often fail to converge on flat regions of the PES or with low-magnitude imaginary modes, making them impractical for large-scale studies.

**Figure 1:**
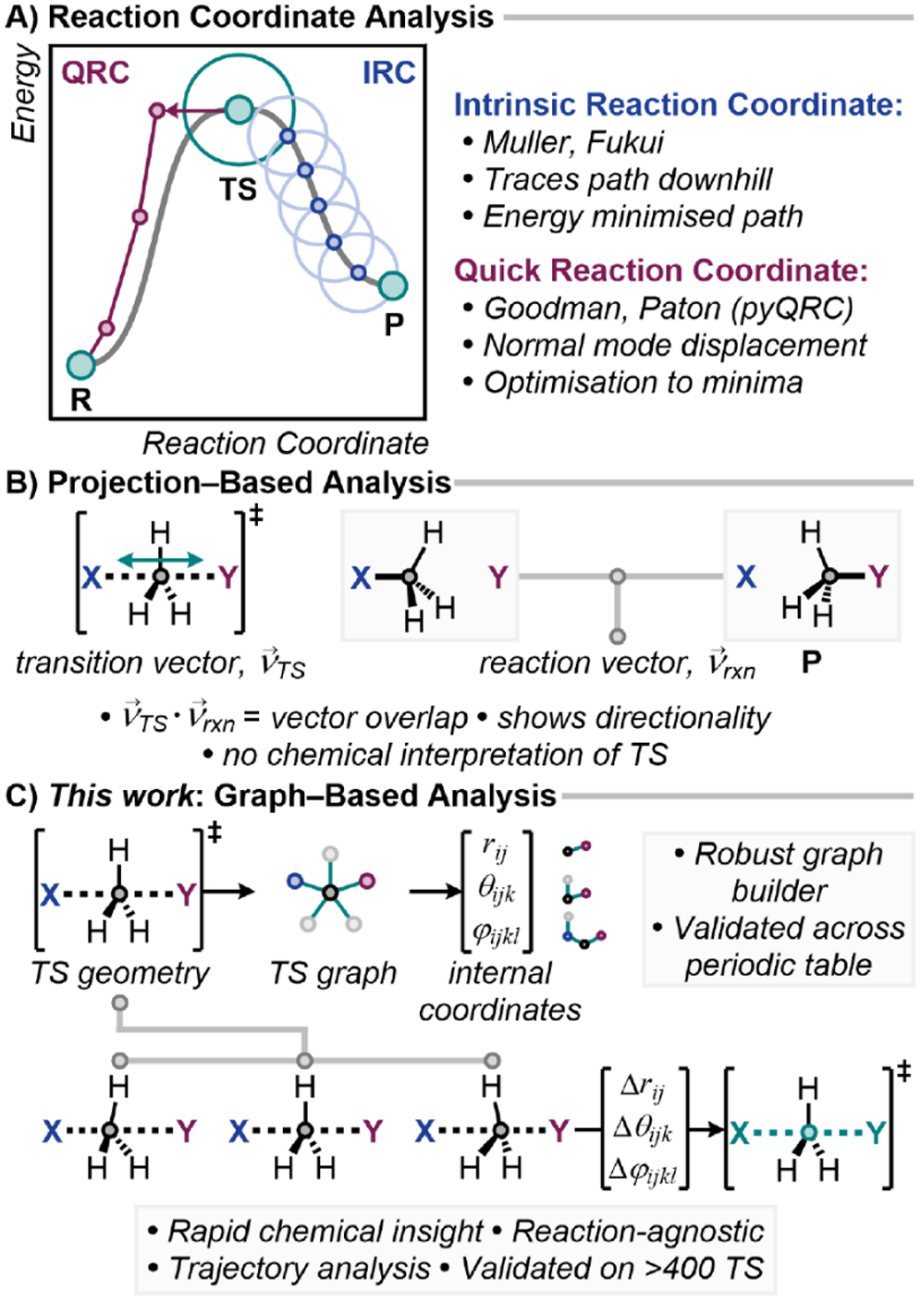
A) Reaction coordinate IRC and QRC transition state validation. B) Normal-mode projection overlap. C) *This work*: TS-centric graph-based internal coordinate analysis.

Quick Reaction Coordinates (QRC), introduced by Goodman and Silva,^4^ offer a quick alternative by geometrically displacing the TS along each direction of the imaginary mode, generating *pre-* and *post-*TS structures, which are then optimized to adjacent local minima (Figure 1A). This approach is widely used to verify connectivity to adjacent minima across organic and organometallic transformations, assisted by the availability of normal-mode displacement in various software packages, including Avogadro,^5^ Molden,^6^ GaussView,^7^ ORCA,^8^ and tools such as pyQRC^9^ and CCLIB.^10^ QRC calculations avoid the expense of IRC calculations by requiring only a single standard geometry optimization in each direction rather than constrained path-following. While this makes QRC simple and reliable across TSs, the cost still scales poorly for validating thousands of TSs, limiting its practicality for high-throughput workflows. The validation bottleneck is increasingly relevant as automated tools such as autoDE,^11^ AARON,^12^ and TS-tools^13^ streamline TS searches, and multilevel workflows that locate TS at a lower level of theory (e.g., semiempirical GFN2-xTB^14^) before refinement at DFT generate large numbers of candidate structures requiring validation. Double-ended methods such as QST2,^15^ NEB,^16^ or FSM^17^ help direct the TS search but still require formal verification that the TS connects the intended reactant and product. Advances in machine learning have driven the production of large-scale data sets, but these often avoid exploring transition states because validation remains computationally complex (e.g., OMol25 ^18^). Some workflows instead adopt a low-cost approach to TS validation by projecting the imaginary mode onto a reaction vector that connects the reactant and product structures.^19,20^ This checks whether the TS mode is broadly aligned with the expected transformation but does not identify which bonds form or break and may mask multistep rearrangements that partially overlap with the expected reaction vector (Figure 1B).

Quantum chemistry calculations naturally produce molecular structures as Cartesian coordinates, but a chemically meaningful interpretation requires molecular graphs and internal coordinates. Molecular graphs and adjacency matrices are widely employed in cheminformatics and machine learning workflows, often using RDKit. Existing graph builders (xyz2 mol^21^ and single-metal extension xyz2 mol_tm^22^) are integrated into RDKit,^23^ using distance-based connectivity and Hückel calculations to reliably assign bond orders for equilibrium structures.^24^ These methods are not designed for transition states and can fail to produce molecular graphs. Partially formed or broken bonds fall outside bonding ranges, formal charge assignments become ambiguous, standard valence rules are violated at atoms involved in bond changes, and multimetal coordination patterns are left unresolved. Automated TS analysis requires a robust graph construction method that can handle nonequilibrium geometries to translate vibrational displacements into changes in chemically meaningful internal coordinates.

Here we present graphRC, a lightweight Python package for automated transition state analysis using internal coordinate tracking across both normal-mode displacements and reaction trajectories (Figure 1C). Unlike projection-based methods that only consider correlation with a specified reaction pathway, graphRC combines robust graph construction with vibrational analysis to characterize the behavior of the TS imaginary mode. The package accepts standard computational output files (including ORCA and Gaussian) containing TS geometries and Hessians for normal-mode projection or reaction coordinate trajectories from IRC or QRC calculations. Molecular graphs are constructed using xyzgraph,^25^ which integrates flexible geometric validation and valence-charge optimization, producing chemically accurate connectivity for both ground-state and transition-state geometries. Building on this framework, graphRC maps normal-mode displacement and reaction coordinate trajectories into internal coordinates, enabling the rapid identification of bond, angle, and dihedral changes along the reaction coordinate without prior knowledge of the reactant or product structures. This reaction-agnostic approach delivers a chemically valid interpretation of the TS mode at a computational cost orders of magnitude lower than that of both IRC and QRC calculations. Structured outputs enable programmatic verification and complement reaction coordinate approaches, offering straightforward integration into high-throughput automated workflows. The package is openly available on GitHub (https://github.com/aligfellow/graphRC.git), pip-installable and free from heavy dependencies. In the following sections, we outline the methodology and validate the performance across diverse examples.

## Methodology

Transition state analysis is performed in internal coordinates, using graph-based connectivity from xyzgraph (https://github.com/aligfellow/xyzgraph.git).^25^ graphRC offers CLI and Python API options for analyzing normal-mode displacement and IRC or QRC trajectories. The workflow is outlined below.

### Molecular Graph Construction

Molecular graph construction is performed using xyzgraph,^25^ which employs a two-pass distance-based approach (Figure 2). The first pass establishes baseline connectivity using van der Waals radii^26^ with thresholds varying by bond type (0.38–0.7 × the sum of vdW radii), applying tighter criteria for covalent bonds and looser ones for metal–ligand interactions. Bonds with interatomic distances well below the threshold (distance <0.6 × threshold) are accepted directly, while longer bonds undergo geometric validation to reject chemically invalid connectivity. Validation includes acute angle criteria (<15° for metals, <30° for nonmetals) and ring diagonal checks using distance ratios to prevent the formation of artifactual three-membered and cross-linked rings. For transition state geometries, a global threshold scaling parameter uniformly extends all distance criteria in a second pass (1.4 × default bonding thresholds), enabling detection of elongated bonds, while geometric validation maintains chemically reasonable connectivity.

**Figure 2:**
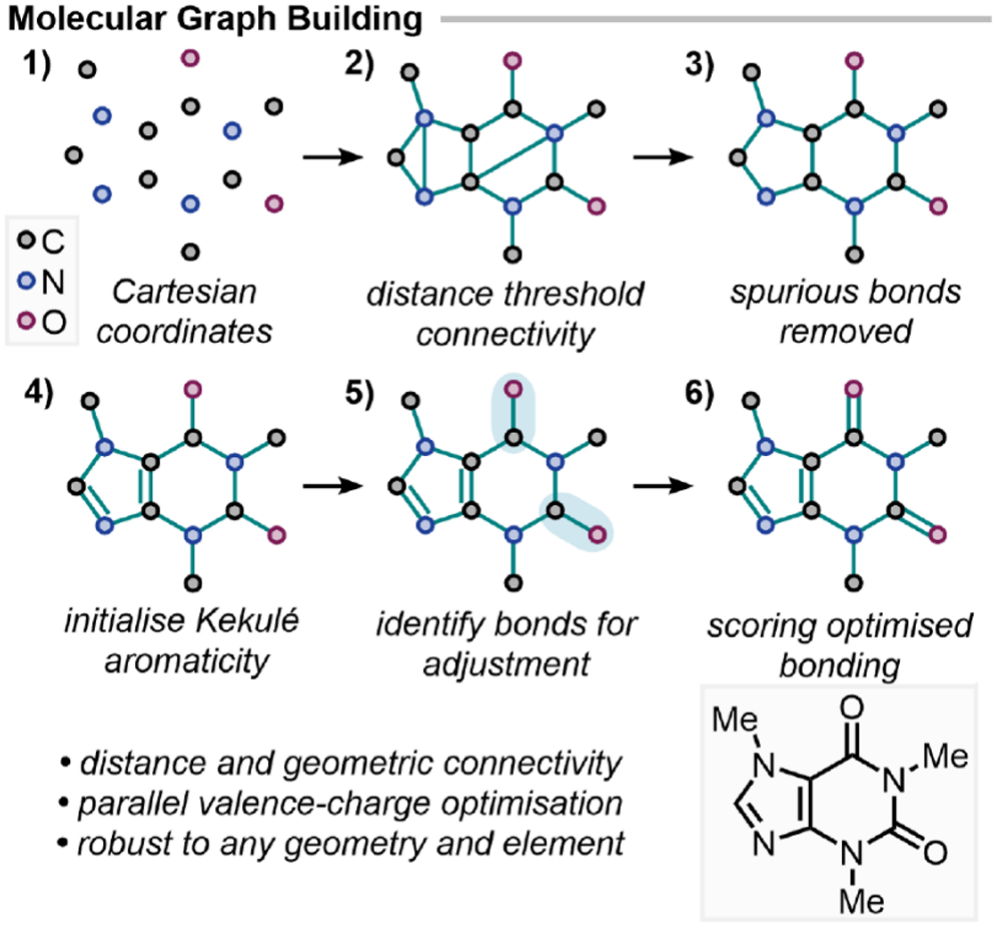
Molecular graph construction workflow using xyzgraph. Geometric validation filters spurious connectivity, followed by parallel valence-charge optimization. The method applies across all elements and produces graph connectivity for any input geometry, allowing for conversion to a set of nonredundant internal coordinates.

Bond orders are optimized iteratively, first beginning with Kekulé initialization for aromatic rings (alternating single and double bonds) to reduce the number of iterations in the optimization process. At each step, bonds with the worst valence description are selected for bond order adjustment (single ↔ double ↔ triple). Changes that minimize formal charges and satisfy valence requirements are prioritized. Multiple candidate bonding patterns are explored simultaneously to avoid convergence to local minima. Metal coordination is handled by fixing metal–ligand bond orders to one throughout the optimization. The bonding pattern of the ligand sphere is then optimized to assign dative or covalent character to the metal–ligand bonds. Finally, the Kekulé aromaticity is converted to aromatic bond orders (1.5) for a uniform representation across aromatic rings.

### Vibrational Analysis

#### 1) File Parsing

The workflow shown in Figure 3 begins either by performing normal-mode displacement on a transition state geometry or by reading an IRC (or QRC) trajectory. Computational output files (from packages such as ORCA^8^ and Gaussian^7^) are parsed using CCLIB^10^ to extract vibrational data and generate a vibrational trajectory.

#### 2) Internal Coordinate Generation

Molecular graphs are constructed from the transition state Cartesian coordinates using xyzgraph. From the graph topology, a set of internal coordinates (bonds, angles, and dihedrals) is generated. Optionally, graphs from reactant and product structures can be used to augment the internal coordinate set, ensuring that key bond coordinates are not missed in the extended TS graph.

#### 3) Trajectory Comparison and Internal Changes

Two frames are selected from the trajectory, either the maximally geometrically diverse (largest pairwise RMSD) or the first and last frames. Internal coordinates are evaluated at each frame, and the changes in bond lengths, angles, and dihedrals are compared.

#### 4) Filtering Correlated Motion

Internal coordinate changes are organized hierarchically to separate primary and coupled motion. Angles and dihedrals involving atoms with changing bonds are filtered out, as these variations are a geometric consequence of the bond change. Equivalent dihedrals that share a rotational axis are filtered to ensure independent rotational characterization, retaining the dihedral defined by the heaviest atoms.

#### 5) Threshold Screening

Default thresholds are applied to detect meaningful changes: 0.4 Å for bonds, 10° for angles, and 20° for dihedrals (initially assigned empirically and validated systematically, *vide infra*). If no changes are detected, thresholds are reduced by 50% to accommodate low-magnitude modes such as hindered rotations or heavy-atom motion. Correlated proton transfers are captured with a secondary reduced threshold, ensuring that each hydrogen-containing bond-breaking event has a corresponding bond-making event, where appropriate.

#### 6) Structured Output

Results are returned as structured dictionaries containing internal coordinate changes, mode characterization, graph objects, and file paths. This structured output enables programmatic TS validation in high-throughput workflows. Displaced structures can also be generated for subsequent QRC analysis (*c.f.* pyQRC).

**Figure 3:**
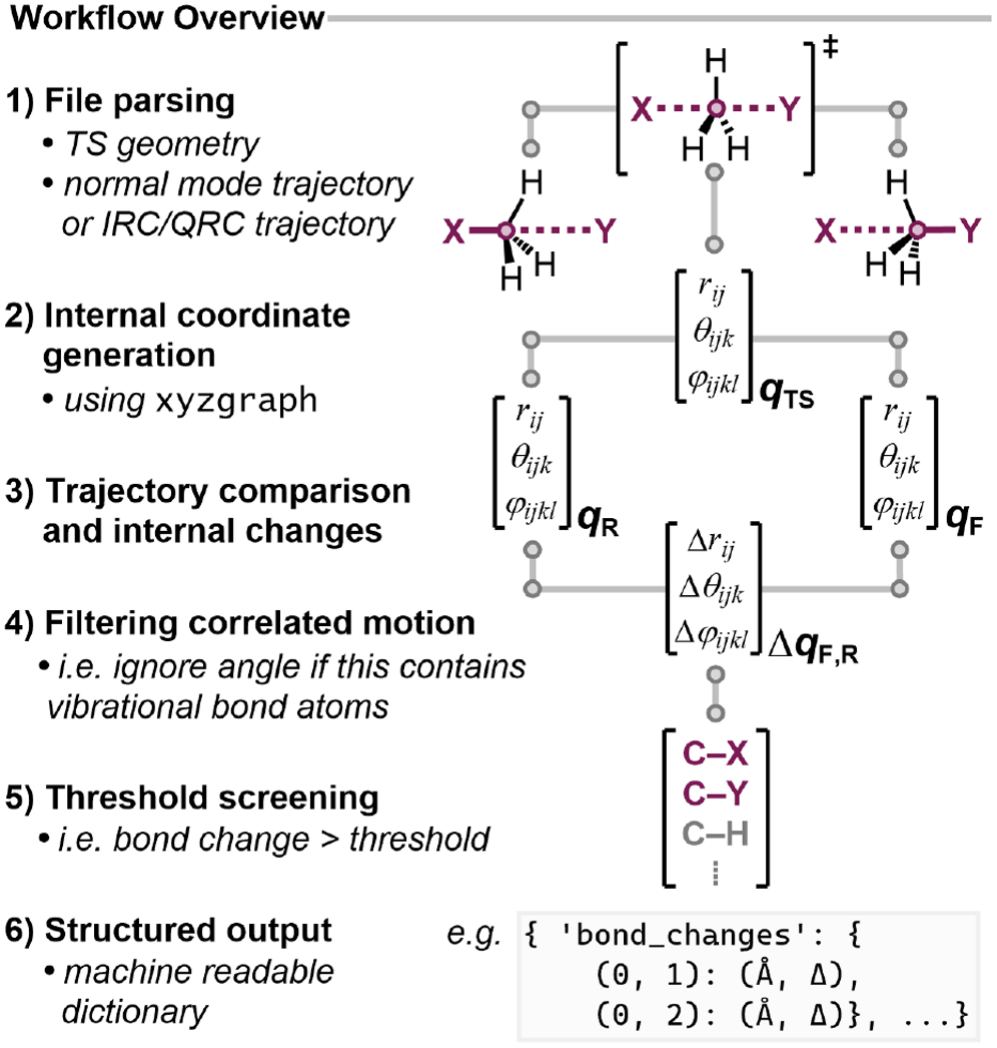
Overview of the workflow employed in this work. *q* is the internal coordinate vector evaluated at the TS and in the forward (F) and reverse (R) directions.

## Results and Discussion

### Graph Building

To ensure a reliable translation from Cartesian into internal coordinates, we first validated the molecular graph-building approach across diverse chemical space. Performance was evaluated using two computational data sets that provide QM-derived bond orders. The first was the GMTKN55 data set, developed by Goerigk and Grimme,^27^ containing 2346 geometries spanning a broad range of organic and main group chemistries designed for functional benchmarking. Because we require a consistent graph-building procedure across the whole periodic table, we also extended the validation to include a stratified sample of 2500 organometallic complexes from the tmQM data sets by Balcells.^28^

Our method successfully generated valid molecular graphs for all of the 4846 structures, spanning 61 elements and including 490 element-pair bond types. Graph connectivity closely agrees with DFT-derived bond connectivity, achieving 98.4% detection, even across exotic bonding environments (Table S1). Performance is comparable to established tools such as RDKit (xyz2mol) and xyz2mol_tm, with 99.8% agreement on GMTKN55 and 97.1% on tmQM, slightly reduced due to the detection of more M–L bonds with xyzgraph. These differences are strongly influenced by the chosen bond order cutoff for single bond definitions (Figures S1–S4). At a bond order cutoff of 0.2, we examined the bonding performance in more detail (Figure 4A). This shows extremely high accuracy for a wide range of bonding types, with some reduced accuracy for unusual bonding patterns of *s*-group metals. The comparatively low performance of C–C (F1 score ≈ 80%) is an artifact of the chosen bond order cutoff, and xyzgraph is consistent with the performance of xyz2 mol (see the SI).

**Figure 4:**
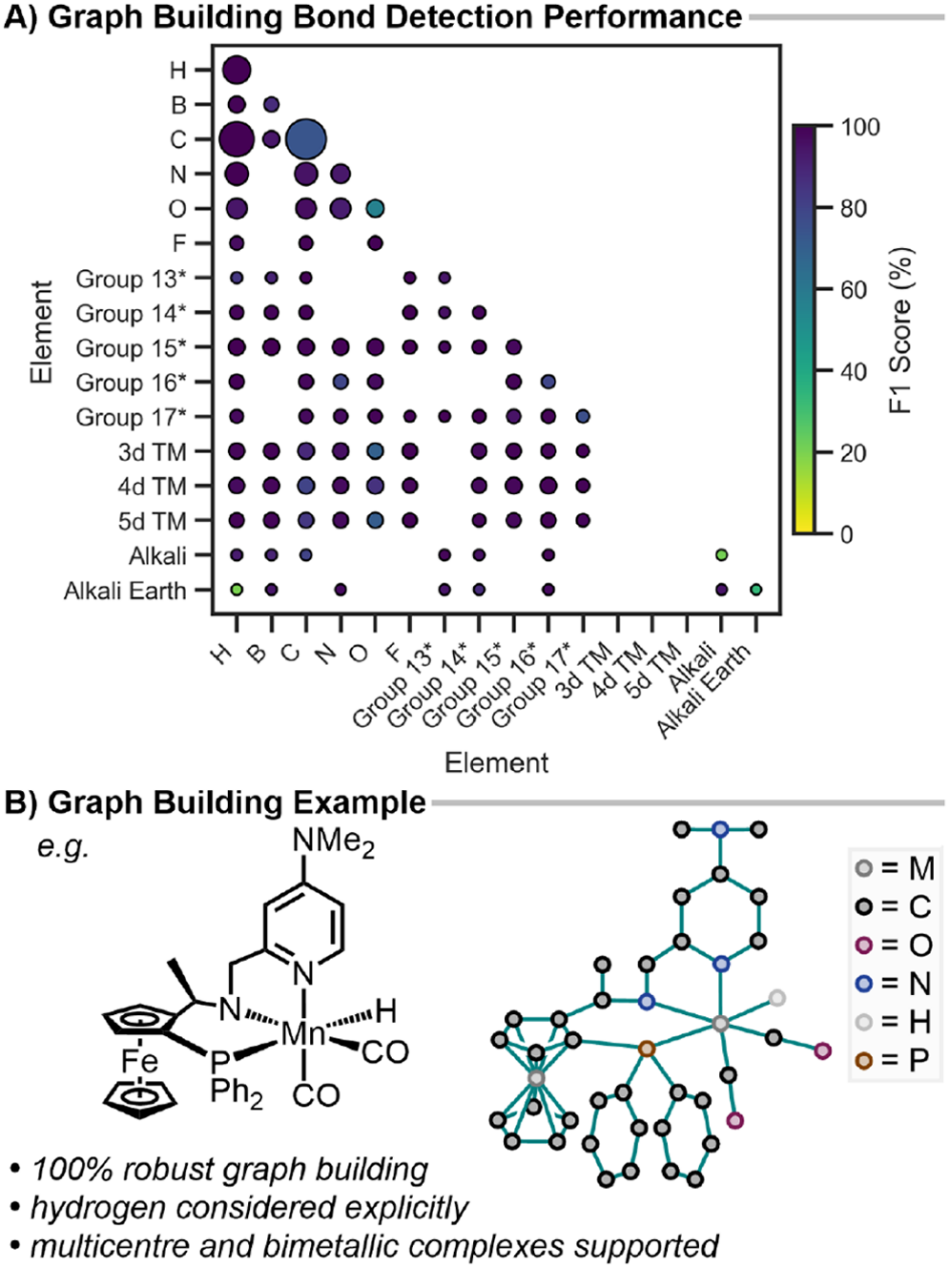
A) Element pair performance (F1 score %) of graph building relative to DFT-derived connectivity using a 0.2 bond order cutoff. Heavy *p*-group elements are grouped for the sake of clarity. An asterisk (*) denotes that these groupings exclude first-row *p*-block elements (which are shown individually). Marker size is proportional to the frequency of the element-pair, colored by F1 score. B) Example of molecular graph building for a bimetallic metal hydride complex. The graph is schematically drawn with nodes (atoms) as dots and detected edges (bonds) as lines. C–H) hydrogens are hidden for clarity.

Overall, these results demonstrate that the graph-building procedure is robust across the entire periodic table and provides a reliable foundation for internal coordinate analysis. A schematic example of the molecular graph built from a bimetallic hydridic complex with multidentate bonding is shown in Figure 4B, with further examples in Figure S6.

### Development of Vibrational Analysis

Having established a robust internal coordinate approach, we next evaluate the vibrational analysis workflow using four simple and well-known transition state examples, including dihedral rotation, pyramidal inversion, and concerted S_*N*_2 reactions (Figure 5). For these small systems, visual inspection of the normal-mode displacement is sufficient for validation, given the limited number of internal coordinates and well-defined transformations. Automated analysis reproduced these results with full accuracy, identifying the relevant bond, angle, and dihedral internal coordinate changes. By incorporating elongated bonds into a set of chemically valid internal coordinates, the method captures connectivity at the nonequilibrium TS geometry, allowing for identification of the TS mode. Postprocessing angle and dihedral changes characterize pyramidal inversions and bond rotations, including methyl rotations (output examples in the SI).

**Figure 5:**
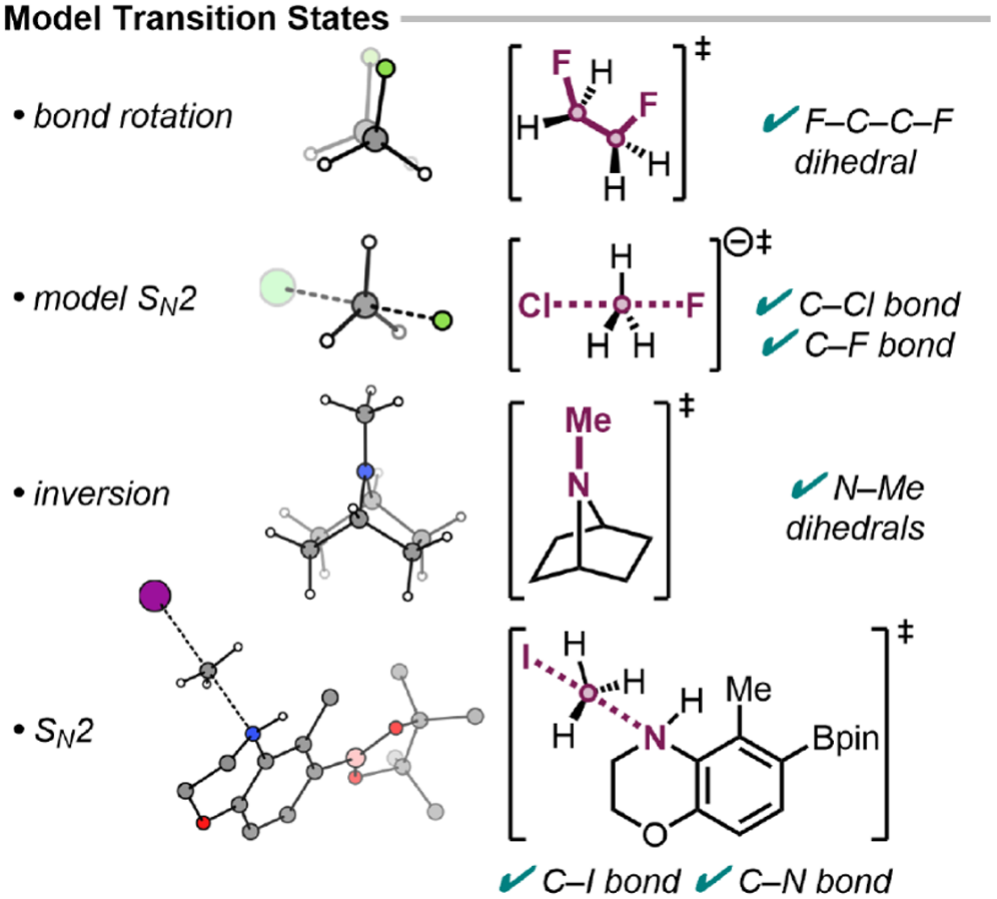
Automated graphRC analysis of model transition states. Visualization of structures using refs. 29,30 .

To demonstrate generalizability, we extended the approach to more complex transition states (Figure 6) from previously published mechanistic studies where IRC or QRC validation data were available. These examples span a wide range of chemical transformations and structural motifs, presenting unique challenges for both vibrational analysis and graph building, with an order of magnitude larger internal coordinate representation than the model systems. For instance, the chiral phosphoric acid-catalyzed Nazarov cyclization involves a proton transfer-initiated cyclization, with three coupled bond changes in the TS. The BIMP-catalyzed (bifunctional iminophosphorane organocatalyst) [2,3]-rearrangement features a “loose” transition state with an asynchronous C–C bond formation, where the degree of asynchronicity is sensitive to the choice of density functional.^31,32,35^

**Figure 6:**
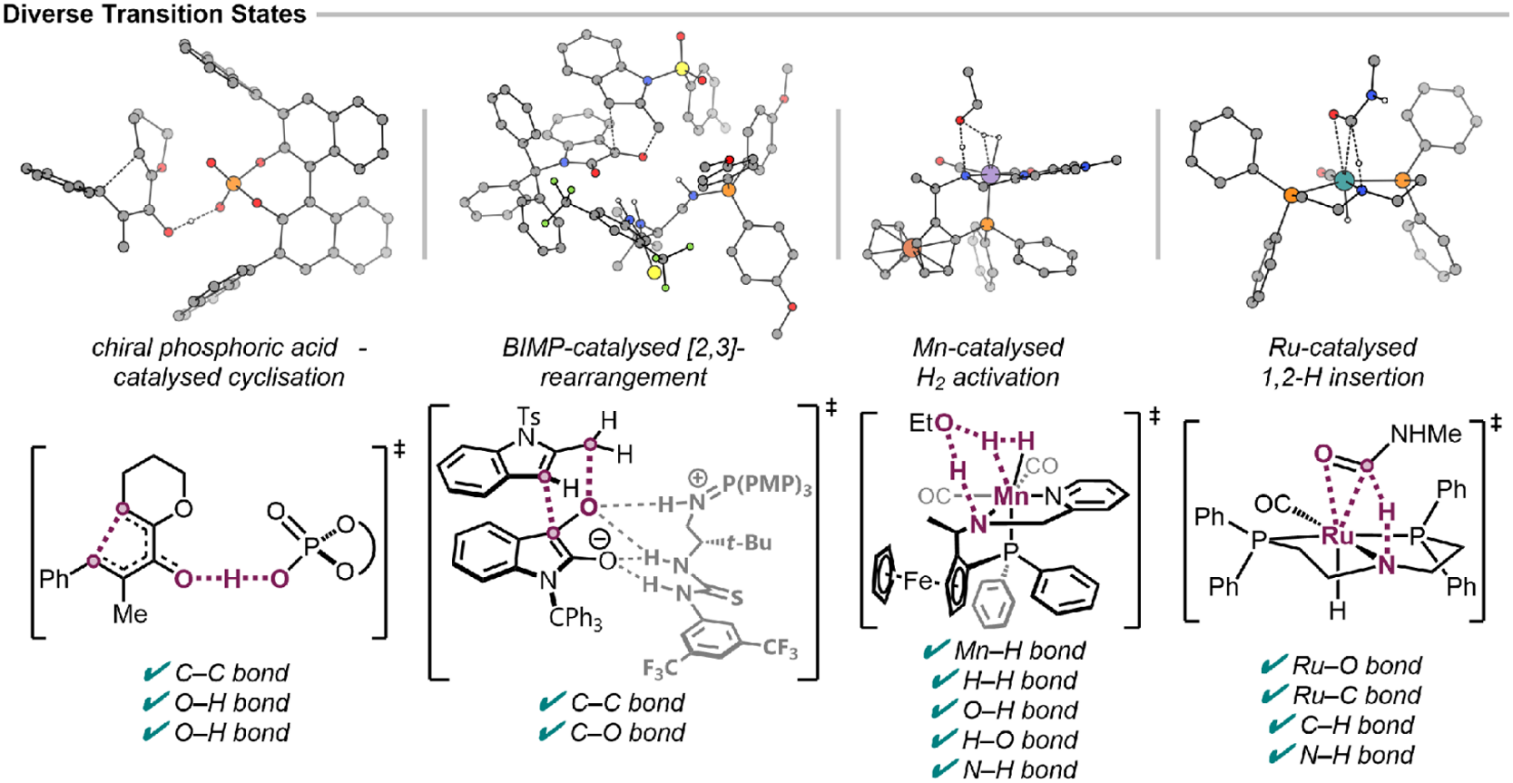
Examples of the graphRC performance on IRC-verified transition states. Each TS is fully characterized by the automated analysis with full detection of the internal coordinate changes and no false positive detections (see also Figures S7–S10).^31–34^

Organometallic systems introduce further complexity for graph building. Unlike organic molecules, where bonding follows well-defined valence rules, transition metal complexes exhibit variable coordination numbers, oxidation states, and binding modes. These include nonclassical binding modes such as η^*n*^-coordination where a ligand binds through *n* contiguous atoms, agostic interactions (weak C–H···M coordination), and multicenter bonding, including bridging hydrides. These arrangements lead to interatomic distances and angles outside standard bonding thresholds and violate valence limits. These challenges are compounded in transition states, where metal–ligand bonds may be partially formed or broken.

The examples studied here test these challenges directly. The Mn-catalyzed H_2_ activation involves an η^2^–H_2_ complex with a ferrocene ligand, alongside five simultaneous bond changes in the TS.^33^ The graph builder accurately represents the chemical structure without the introduction of spurious M–L bonds. The Ru-catalyzed 1,2-hydrogen insertion TS involves 3-membered rings with an η^2^–C=O arrangement and a proton transfer that occurs late in the reaction coordinate, with only a small displacement along the imaginary mode eigenvector (Figure S10).^34^ In both examples, the graph builder accurately represents the coordination environment, achieving full accuracy for vibrational analysis.

Additional validation examples exhibit greater structural diversity (Figures S11–S18). Atropisomeric hindered rotations are extremely challenging to characterize using IRC calculations due to low-magnitude imaginary modes.^36^ As a result, normal-mode displacement produces only a small Cartesian displacement, and we apply a secondary round of 50% relaxed thresholds, which leads to a fully consistent characterization of bond rotations in the TS. Spirocyclic transition state geometries present another unique challenge, with a 5-membered ring attached to a 4-membered ring. This was fully characterized without introducing spurious cross-linking that would lead to the wrong TS mode identification.^37^

These 16 TS examples are summarized in Table 1, covering proton transfers, C–C and C–X bond formations and cleavage, ring rearrangements, cyclizations, hindered bond rotations, and pyramidal inversions across organic and organometallic transitions. Across all examples, the method achieved 100% detection of relevant internal coordinate changes with zero false positives, verified against both IRC and QRC connectivity.

**Table 1:** Sixteen Transition State Examples Analyzed in Detail^a^

TS	Transformation	Class	Complexity	Features	Accuracy	Validation
dihedral rotation	F–C–C–F rotation	model	•◦◦ (1)	low-barrier torsion	100%	VI
model S_*N*_2	C–Cl break + C–F form	model	•◦◦ (2)	concerted	100%	VI
pyramidal inversion	N inversion	model	•◦◦ (1)	atom inversion	100%	VI
S_*N*_2	C–I break + C–N form	organic	•◦◦ (2)	concerted	100%	VI
Nazarov cyclization^31^	C–C ring close + O–H–O *pt*	organic	••◦ (3)	proton transfer + ring close	100%	IRC
[2,3]-rearrangement^32^	C–O break + C–C form	organic	••◦ (2)	“loose” TS, asynchronous	100%	IRC
Mn–H_2_ activation^33^	H–H break + N–H–O *pt*	organo-metallic	••• (5)	η^2^-H_2_ bonding + solvent	100%	IRC
Mn-catalyzed reduction^33^	Mn–H–C hydride transfer	organo-metallic	••◦ (2)	metal binding	100%	IRC
Aza-Morita-Baylis-Hillman^38^	C–H–N *pt*	organic	•◦◦ (2)	proton transfer	100%	IRC
Ru-catalyzed insertion^34^	M–L rearr. + N–H–C *pt*	organo-metallic	••• (4)	M–L 3-membered ring + rearrangement	100%	IRC
thia-Michael addition^36^	C–S form	organic	•◦◦ (1)	sulfur nucleophile	100%	IRC
atropisomeric rotation^36^	hindered bond rotation	organic	••◦ (2)	low-frequency mode	100%	QRC
Ru methylamine elimination^34^	C–N break + N–H–N *pt*	organo-metallic	••◦ (3)	asynchronous	100%	QRC
isothiourea cyclization^36^	C–N ring close	organic	•◦◦ (1)	ring closure	100%	QRC
spirocyclic Nuc-attack^37^	C–N form	organic	••◦ (1)	small rings	100%	QRC
spirocyclic ring opening^37^	C–O break	organic	••◦ (1)	small rings	100%	QRC

a These TSs were selected from a combination of previous mechanistic work and broader literature examples to provide a diverse and challenging set, including simple model systems and complex organometallic transformations . Complexity is shown as filled dots (•) out of three, with the number of internal coordinate changes in the TS in parentheses. Accuracy is reported as the F1 score (harmonic mean of precision and recall). VI = Visual Inspection; *Pt* = Proton Transfer.

### Scalability and Threshold Optimization

Following validation, we assessed the computational efficiency, which is critical for high-throughput workflows. As an illustrative comparison, the 172-atom BIMP transition state required ∼151 h of wall time for the IRC calculation on 16 cores (≈2410 core hours), while a QRC calculation required ∼44 h on 32 cores (≈1398 core hours). In contrast, graphRC analysis of the optimized TS completed in roughly 2 s on a single core, approximately 6 orders of magnitude faster, while retaining full accuracy for bond detection. This difference illustrates the scalability of this approach: as IRC and QRC calculations become prohibitively expensive, the cost of graphRC remains negligible (Figure 7).

**Figure 7:**
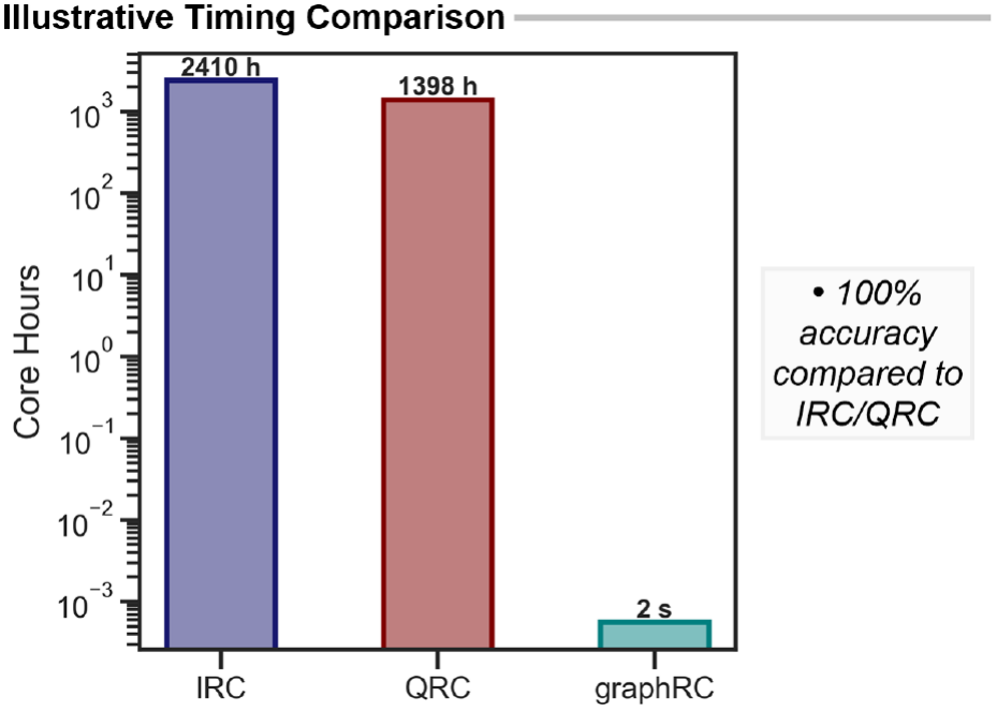
Illustrative comparison of computational time across IRC, QRC, and graphRC methods for a BIMP-catalyzed [2,3]-rearrangement.

A bond detection threshold defines the minimum bond length change required to flag a bond as forming or breaking during the vibrational mode. The magnitude of these changes depends on the displacement amplitude used when projecting the imaginary mode eigenvector onto the TS geometry, as a larger displacement produces larger Cartesian changes. A fixed displacement amplitude is used throughout to produce chemically reasonable displaced structures, and the detection threshold was optimized at this amplitude. Scanning the bond change threshold from 0.1 to 0.5 Å across all 16 examples from Table 1 revealed that 0.4 Å provides an optimal balance, achieving full detection of expected bond changes with zero false positives (Figure 8). Below this value, small-amplitude displacements are incorrectly identified as bond changes, and above it, true bond changes start to be missed. This choice prioritizes precision over exhaustive detection, avoiding overinterpretation of Cartesian displacements for reliable high-throughput analysis. While fully accurate for the examples above, this threshold may miss highly asynchronous bond coordinate changes, where the displacement along the imaginary mode is small (*vide infra*).

**Figure 8:**
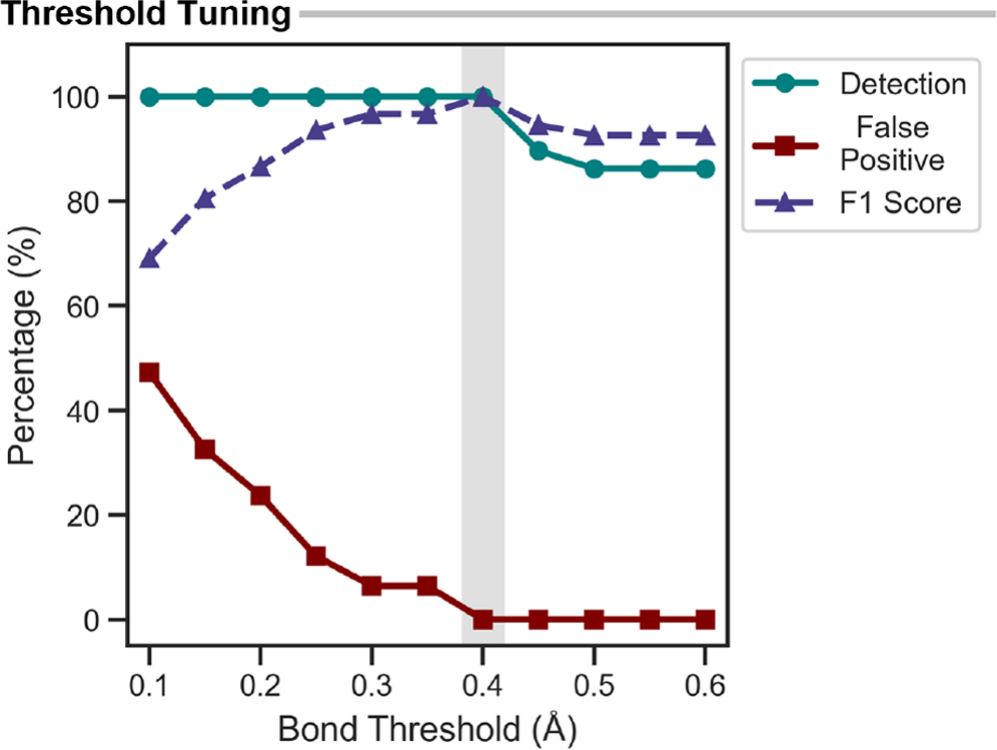
Threshold optimization metrics include detection (% of expected bonds found), false positive (% of spurious detections), and F1 score (%) (balanced measure combining both metrics, expressed as a %). Optimal default threshold highlighted in gray (0.40 Å).

### Limitations

While the optimized threshold performs very well across the examples studied, highly asynchronous or late bond formation can still pose challenges (Figure 9). This reflects the inherent nature of normal-mode analysis, which approximates the transition as a single displacement vector at the saddle point. While this linear projection captures the primary transformation accurately, it cannot fully describe the complex, multidimensional surface of highly asynchronous transition states. To stress-test the methodology, we applied it to two literature examples of strongly asynchronous concerted transformations verified by IRC. In both cases, the automated analysis correctly identifies the primary internal coordinate change but struggles to fully capture the asynchronous changes.

**Figure 9:**
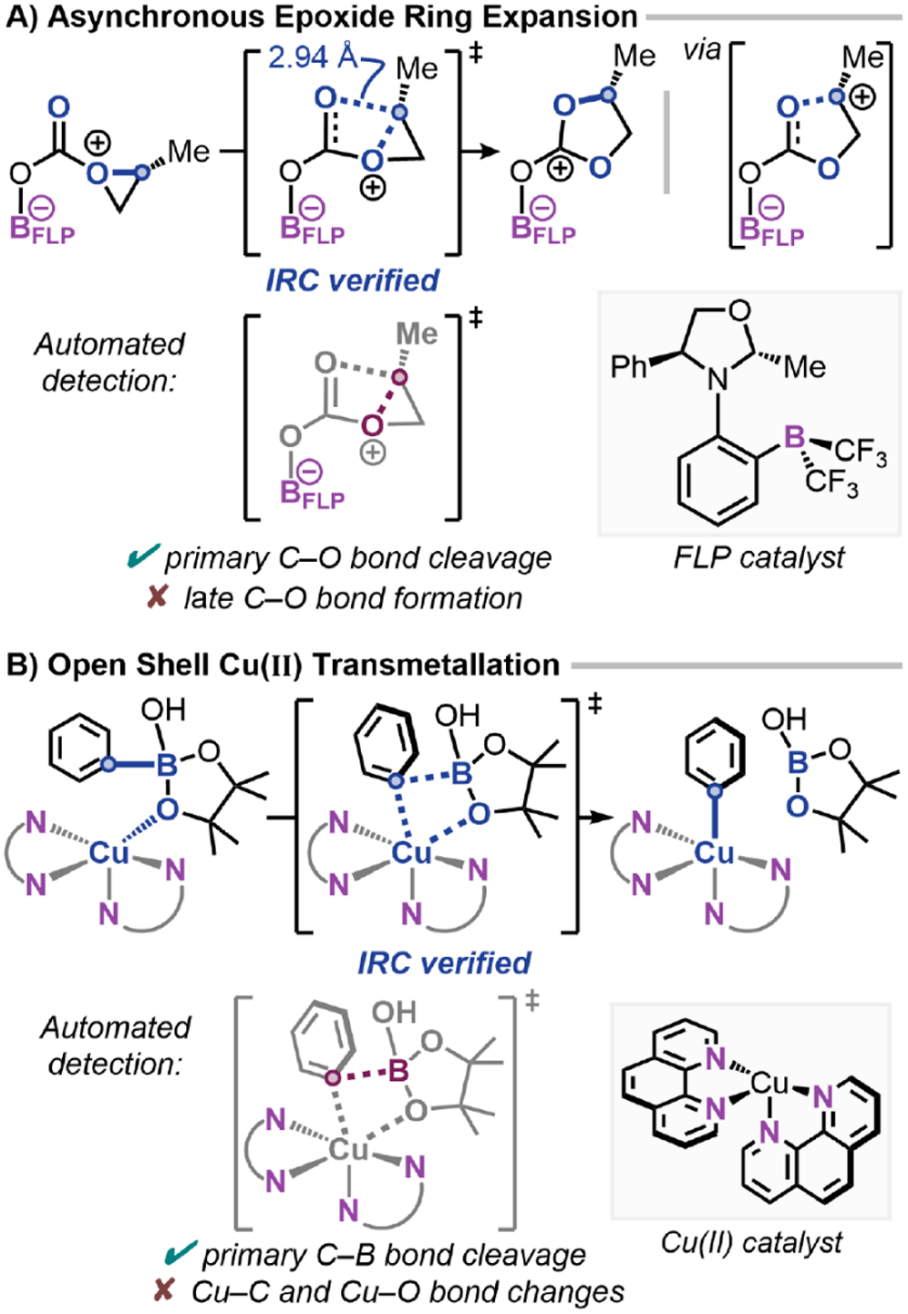
Examples that push the limits of the normal-mode displacement approach compared to the IRC connectivity. A) Epoxide ring expansion.^39^ B) Open-shell Cu­(II) transmetalation.^40^

In the epoxide ring expansion reported by Trujillo et al.,^39^ the analysis correctly identifies the epoxide O–C ring opening but does not detect the strongly asynchronous C–O bond formation in this TS. This bond forms late along the reaction coordinate and has a bond length of 2.9 Å in the TS, beyond the default threshold for constructing the internal coordinate. Relaxing connectivity criteria allows this bond coordinate to be identified; however, this introduces false positive internal coordinate changes that reduce confidence and chemical interpretability. In such cases, we argue that a partial identification of the primary transformation is preferable to overdetection. Analysis of the IRC trajectory fully captures the TS connectivity, capturing both the epoxide ring opening and 5-membered ring closing in the TS mode. Similarly, in the Cu­(II) open-shell transmetalation by Macgregor et al.,^40^ the method detects the primary C–B bond cleavage but misses the Cu–C and Cu–O bond changes unless the threshold is reduced to 0.3 Å.

These cases highlight the limitations of normal-mode analysis, which cannot fully capture asynchronous multidimensional behavior. For application to rapid mechanistic screening and automated workflows, prioritizing the primary transformation is preferable to exhaustive detection that may introduce artifacts. Where asynchronous transitions are present, a short QRC path can complement this approach, enabling an interpretable internal coordinate analysis across IRC and QRC pathways.

### High-Throughput Validation

To further assess the robustness of this approach, we extend the analysis across 395 transition states from reference 41,42 which provides GFN2-xTB^14^ IRC trajectories for transition states mined from the Supporting Information of published studies. These 395 TS contain 952 vibrational bonds, including 35 elements involved in 89 unique bond types (Figure 10).

**Figure 10:**
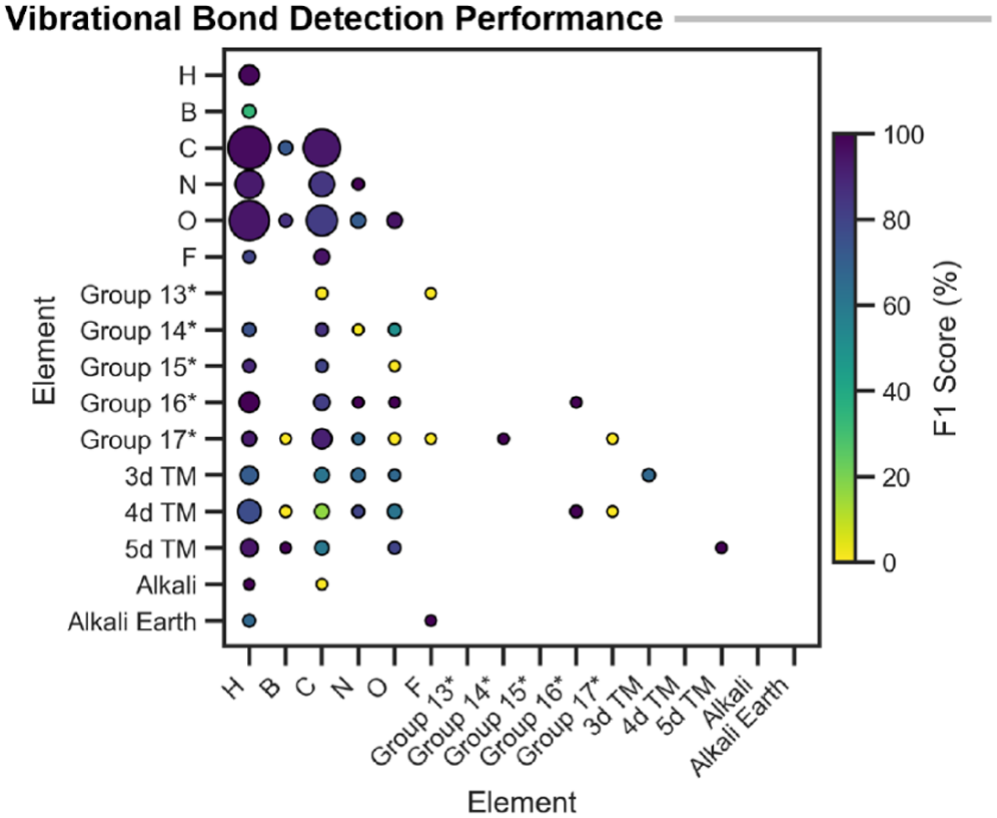
Element pair performance (F1 score %) of automated vibrational analysis relative to GFN2-xTB-derived IRC connectivity. Heavy *p*-group elements are grouped for clarity. An asterisk (*) denotes that these groupings exclude first-row *p*-block elements (which are shown individually). Marker size is proportional to the frequency of the element-pair, colored by F1 score.

Using only normal-mode displacement, the method achieves an F1 score of 88.4% and correctly identifies the primary bond change in 100.0% of transition states compared with full IRC calculations. Across the bond changes identified by IRC, 81.8% are detected while maintaining a high precision of 96.1% (false positive rate of 3.9%). This diverse data set covers a variety of organic, organometallic, and catalytic examples, including proton transfers, Diels–Alder, various M–L rearrangements, and ring opening and closing (20 random examples shown in Figures S19–S22). In more detail, the element-pair performance is shown in Figure 10. Common organic transformations involving O–H, N–H, C–H, C–C, C–O, and C–N dominate and are identified with high accuracy. Organometallic reactivity is also represented, including M–H activation and M–L coordination changes. A reduced performance is observed for boron and some heavier elements; however, this may partly reflect limitations of the GFN2-xTB level of theory. Overall, these 395 TS cover a wide range of relevant chemical reactivity with fully validated IRC bond changes. Extended analysis under relaxed TS criteria increases elemental coverage but includes TS that are not strictly IRC-validated, reducing confidence in the underlying IRC-based ground truth (Figure S25).

Stratifying by the number of bond changes reveals that detection accuracy decreases with increasing complexity (Figure 11A and Table S2). 100.0% of single-bond changes are detected, though detection drops for more complex transformations with >6 bond changes. False positives remain low overall and are negligible for TS with >2 bond changes, though they are slightly more frequent for TS with one bond change event. This behavior reflects the limitation of normal-mode displacement, which uses a single vector projection to approximate a multidimensional transformation. Despite the reduced accuracy for the most complex examples with >6 bond changes, the overall performance is dominated by less complex TS (Figure 11B). The most common category, with two bond changes (44.8% of the data set), achieves 91.8% detection with 97% precision. These metrics confirm that the method maintains high accuracy across the majority of transition states while prioritizing precision over exhaustive detection.

**Figure 11:**
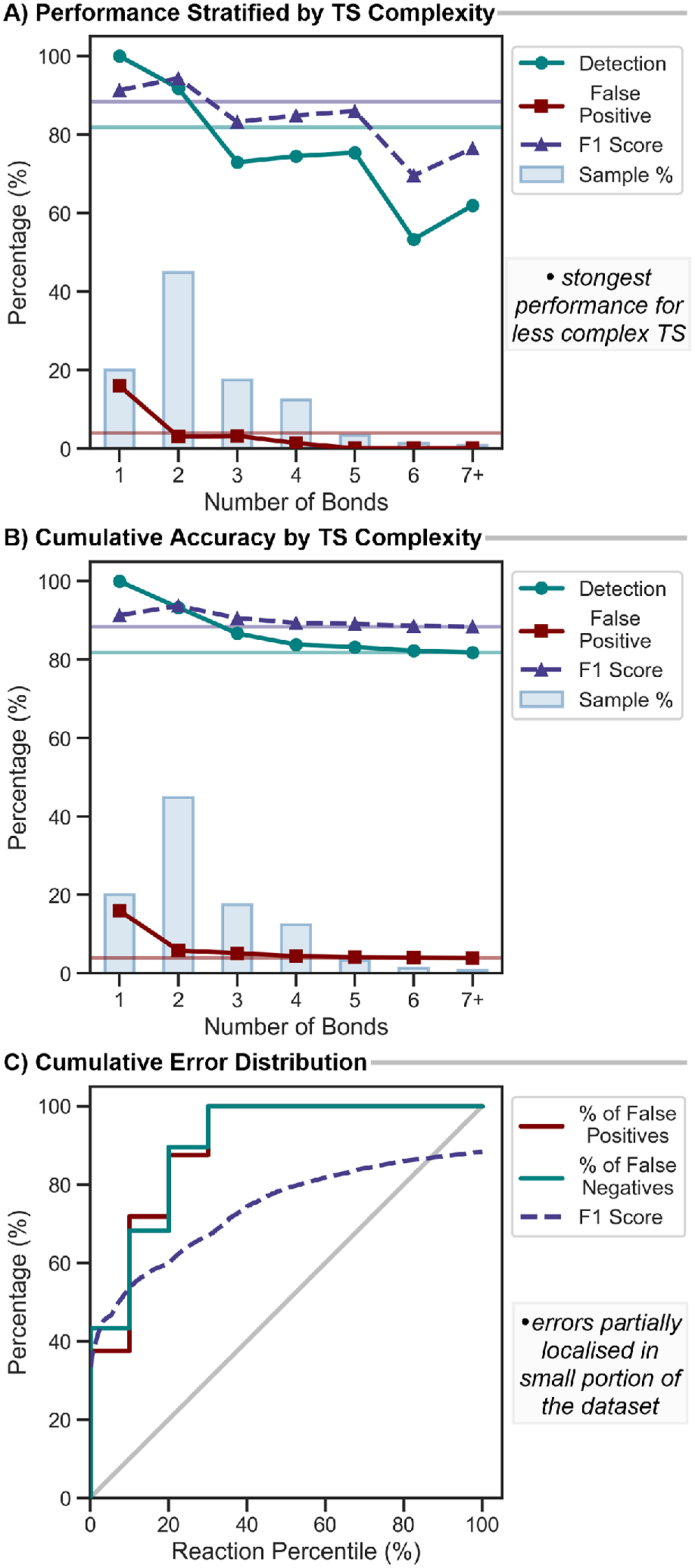
Element-pair performance (F1 score %) compared to GFN2-xTB-derived IRC connectivity. A) Performance metrics stratified by the number of bond changes in each transition state. Average is shown with horizontal lines. Bars show the distribution of the TS complexity. B) Cumulative performance across increasing TS complexity. C) Cumulative error distribution across data set percentiles ranked by F1 score. Stepped lines represent cumulative percentages of false positives and false negatives. F1 score is shown as a rolling average. Identity line shown in gray.

Additionally, we suspect that this reduced performance may be partially related to the GFN2-xTB level of theory used for TS identification and IRC path evaluations. GFN2-xTB can favor more concerted transformations compared to higher levels of theory. To investigate, we examined the error distribution across the data set. Ranking samples by F1 score revealed that the lowest 10% of TS accounted for 42.4% of all errors, including 37.5% of false positives and 43.4% of false negatives (Figure 11C). Inspection of the six TS with F1 scores <50% (Figures S23–S24 ^43–48^ ) showed a mixture of highly asynchronous, complex, multibond rearrangements, which reflect two related limitations. Normal-mode displacement cannot fully capture these transformations (*vide supra*), and the concertedness of these reactions may be exaggerated by the GFN2-xTB level of theory used for TS and IRC evaluation. Exclusion of the 19 worst-performing cases (bottom 5%) yielded 89.1% detection with only 2.4% false positives and an F1 score of 91.5%. These 19 cases alone account for a quarter (24.9%) of the total errors, highlighting that the overall metrics understate the performance of the method for typical cases.

Overall, these results confirm that the approach is appropriate for transition state analysis, with strong performance across a wide range of transformations in complex bonding scenarios. Notably, all 411 examples achieved 100.0% detection of the primary bond transformation, with 96.1% precision across 984 bond change events in 395 high-throughput IRC-validated examples. This implementation offers a rapid tool for mechanistic studies, characterizing transition states, facilitating QRC calculations, and formal analysis of QRC and IRC trajectories. The structured output of the code supports integration into high-throughput campaigns and can be incorporated into feedback loops to refine geometries when secondary imaginary modes, such as methyl rotations, are present. Combined, these features enable scalable, automated TS analysis that reduces the computational cost by orders of magnitude compared with IRC pathways while retaining high accuracy across diverse reaction classes.

## Conclusion

graphRC offers a fast, reliable, and generalizable approach to transition state (TS) analysis, validated across a broad variety of organic and organometallic transformations. Graph-based internal coordinate construction through xyzgraph enables the accurate identification of bond formation, cleavage, rotations, and inversions while avoiding false positives in complex or low-magnitude vibrational modes. We have demonstrated high accuracy in identifying key internal coordinate changes, and its structured outputs make it particularly suited to automated workflows. The practical applications are 3-fold:

(1) **Mechanistic analysis**: Rapid verification of the TS mode prior to formal IRC or QRC calculations. Analysis of nonequilibrium structures with multiple imaginary frequencies. Generation of displaced geometries for eliminating small imaginary modes (*c.f.* pyQRC^9^).
(2) **High-throughput TS campaigns**: A structured output provides machine-readable internal coordinate changes that can be integrated into automated pipelines that make use of atom-mapping or adjacency matrices for programmatic TS verification. These features support screening workflows that terminate upon the successful detection of targeted bond changes, and flexible displacement along vibrational modes supports the elimination of small imaginary frequencies, streamlining TS workflows.
(3) **Reaction coordinate analysis**: Applied to IRC or QRC trajectories, the analysis translates Cartesian changes into chemically intuitive internal coordinate changes that reflect the underlying reaction mechanism. This can be integrated into high-throughput workflows by performing short, partial optimizations of displaced TS structures for enhanced accuracy compared to mode displacement.

Importantly, the tool prioritizes accuracy through careful graph-building logic, avoiding spurious bonds that would introduce meaningless internal coordinate changes. Detection of highly asynchronous transformations remains a challenge due to the limitations of using mode projections; however, a conservative approach ensures that reported changes are chemically meaningful and reliable. We emphasize the importance of IRC and QRC calculations for the true characterization of transition states.^49^ graphRC can also be applied to these trajectories, providing a formal internal coordinate description of the reaction coordinate. This tool is designed to supplement these methods, offering rapid mechanistic analysis and supporting high-throughput scenarios where IRC calculations are unfeasible.

## Supplementary Material



## Data Availability

The graphRC software package is available on GitHub (https://github.com/aligfellow/graphRC.git), v1.3.5. Threshold analysis can be reproduced using scripts in the repository. Further output and usage examples are documented in the GitHub repository. The xyzgraph software package is also available on GitHub (https://github.com/aligfellow/xyzgraph.git), v1.4.8. IRC and QRC data generated in this work are available on Zenodo (https://doi.org/10.5281/zenodo.17877172).

## Notes

Graph building analysis, output examples, IRC and QRC pathways, high-throughput validation workflow and analysis (PDF)
