# Supplementary material for: Graph-Based Internal Coordinate Analysis for Transition State Characterization
Source: J Chem Theory Comput. 2026 Feb 21;22(5):2348–57. doi: 10.1021/acs.jctc.5c02073 (PMC12980719; doi:10.1021/acs.jctc.5c02073)
Supplement: Supplementary file 1 [file ct5c02073_si_001.pdf]

Supporting Information for

Graph-Based Internal Coordinate Analysis for Transition State Characterisation

Alister S. Goodfellow,[*a] and Bao N. Nguyen[a]*

[a] *School of Chemistry, University of Leeds, Woodhouse Lane, Leeds, LS2 9JT, UK.*
E-mail: a.s.goodfellow@leeds.ac.uk

Contents

1 Graph connectivity validation **S2**
- 1.1 Stratified Sampling for `tmQM` . . . S2
- 1.2 Overall Metrics . . . S2
- 1.3 Bond Order Cut-off Sensitivity . . . S2
- 1.4 Element Performance . . . S5
- 1.5 Random Structures from tmQM . . . S6

2 Output Examples **S7**
- 2.1 S_N2 TS CLI output . . . S7
- 2.2 N-inversion TS CLI output . . . S8
- 2.3 BIMP TS CLI output . . . S9

3 Transition State Examples **S10**
- 3.1 IRC verified . . . S10
- 3.2 QRC verified . . . S17

4 High Throughout Validation **S22**
- 4.1 Stratified Performance . . . S22
- 4.2 Random Examples . . . S23
- 4.3 Low Accuracy High-Throughput Examples . . . S27
- 4.4 Relaxed TS Criteria . . . S30

References **S31**

1 Graph connectivity validation

To validate the geometric graph building approach we compare the graph connectivity against DFT bond order connectivity across the GMTKN55[1] and tmQM[2] datasets. Mayer bond orders from PBEh-3c single-point calculations are available for 2346 of the 2462 structures in the GMTKN55 dataset (https://github.com/grimme-lab/GMTKN55.git) and Wiberg bond orders from TPSSh-D3BJ/def2-SVP single-point calculations are available for 108,541 complexes in the tmQM dataset (https://github.com/uiocompcat/tmQM.git).

1.1 Stratified Sampling for tmQM

Molecules were grouped by metal and stratified by coordination number (CN), charge, and multiplicity. Sampling quotas for each metal were proportional to the distribution in the full dataset. Random samples were taken from each combination of CN, charge, and multiplicity.

1.2 Overall Metrics

Table S1: Performance of graph construction across benchmark datasets compared to DFT connectivity with bond order > 0.2 and existing tools. Detection, false positives and F1 score are reported in %.

Dataset	Comparison Method	Structures	True Positives	False Positives	F1 Score
GMTKN55 + tmQM	DFT	4846	98.4	0.4	98.8
GMTKN55	DFT	2346	98.1	0.3	98.6
tmQM	DFT	2500	98.6	0.5	99.0
GMTKN55	RDKit (xyz2mol)	1860	99.8	0.2	99.8
tmQM	xyz2mol_tm	2220[a]	99.9	5.9	97.1

[a] Due to the reordering of atom indices, the resulting molecular graphs from xyzgraph and xyz2mol_tm are topologically aligned to compare atom and bond indices accurately. 2220/2500 reflects both the success of xyz2mol_tm and the ability to accurately perform partial graph matching.

1.3 Bond Order Cut-off Sensitivity

Performance against DFT is sensitive to the choice of bond order cut-off. Weak bonds, including metal-ligand coordination and strong non-covalent interactions can fall in a similar range. **Figures** S1–S4 illustrate how varying the cut-off changes the xyzgraph performance. At higher thresholds, true weak M−L bonds are excluded from the reference graph, which increases the error count as these bonds remain in the geometric detection. Conversely, raising the threshold removes spurious M−L connections that arise from small bond orders between adjacent atoms, reducing false positives in the ground truth. We chose a BO threshold of 0.2 to balance these effects, and present a conservative estimate of bond detection performance.

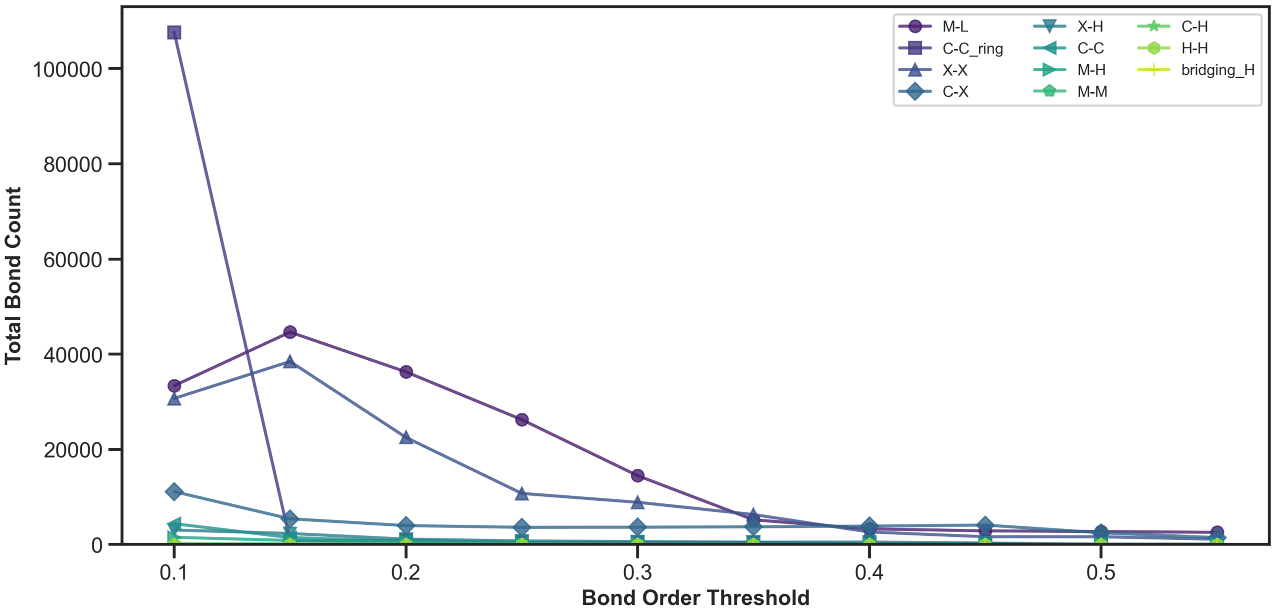

Figure S1: Total number of bonds detected by DFT as ground truth across different BO threshold values.

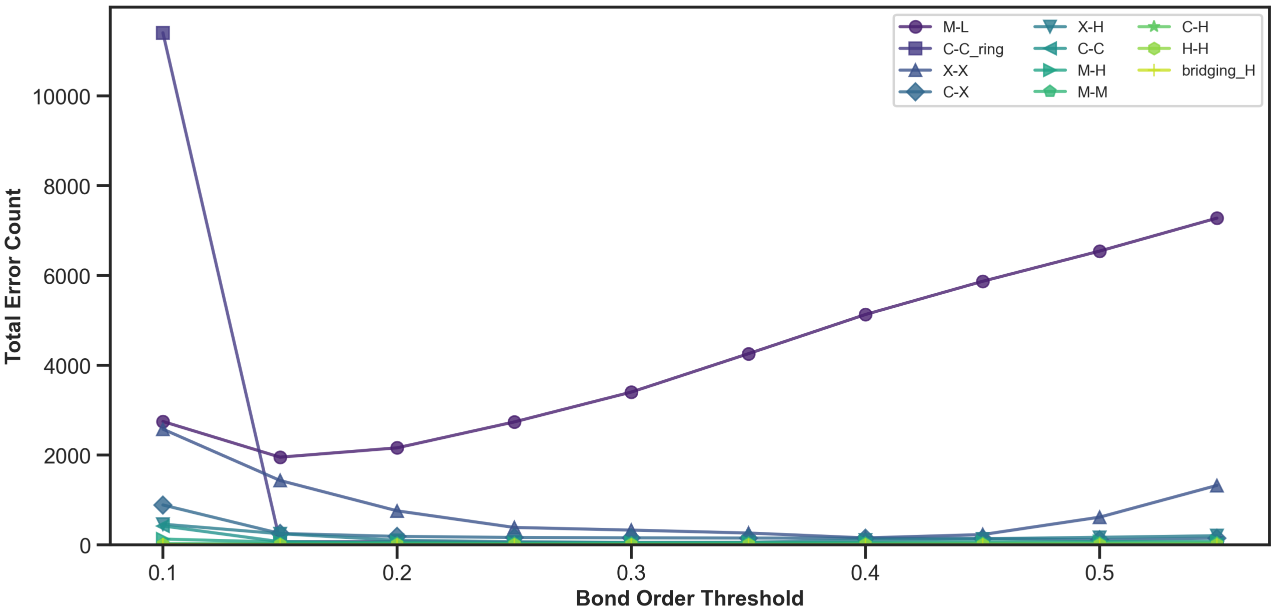

Figure S2: Total error count of bond detection compared to DFT as ground truth across different BO threshold values.

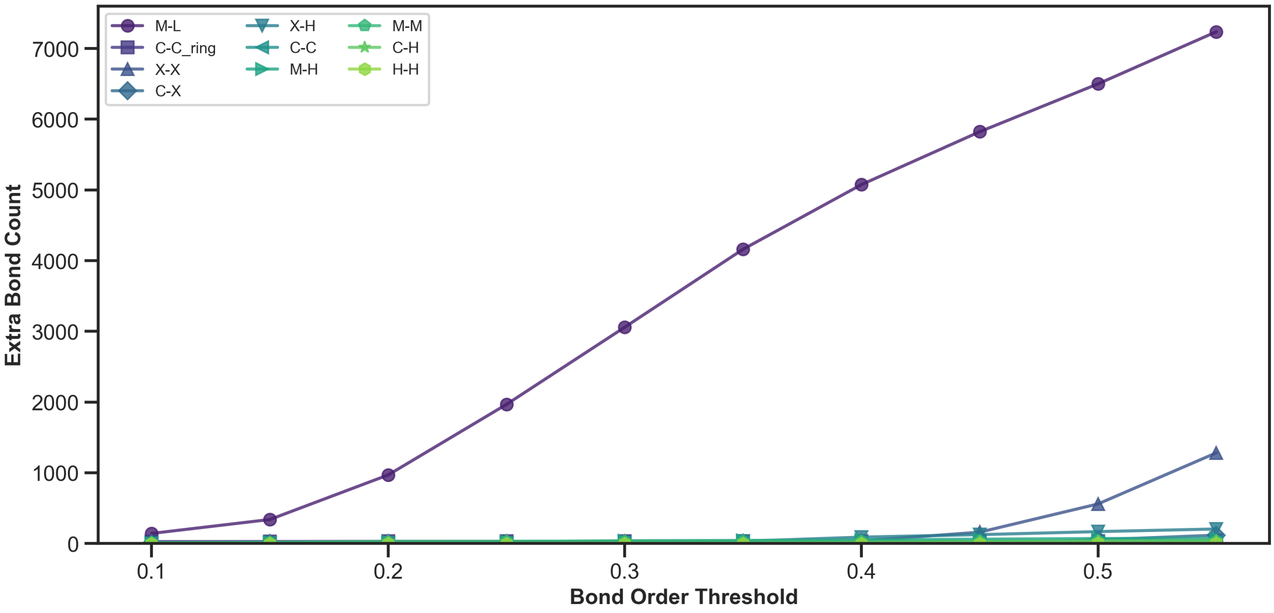

Figure S3: Total false positive count of bonds compared to DFT as ground truth across different BO threshold values.

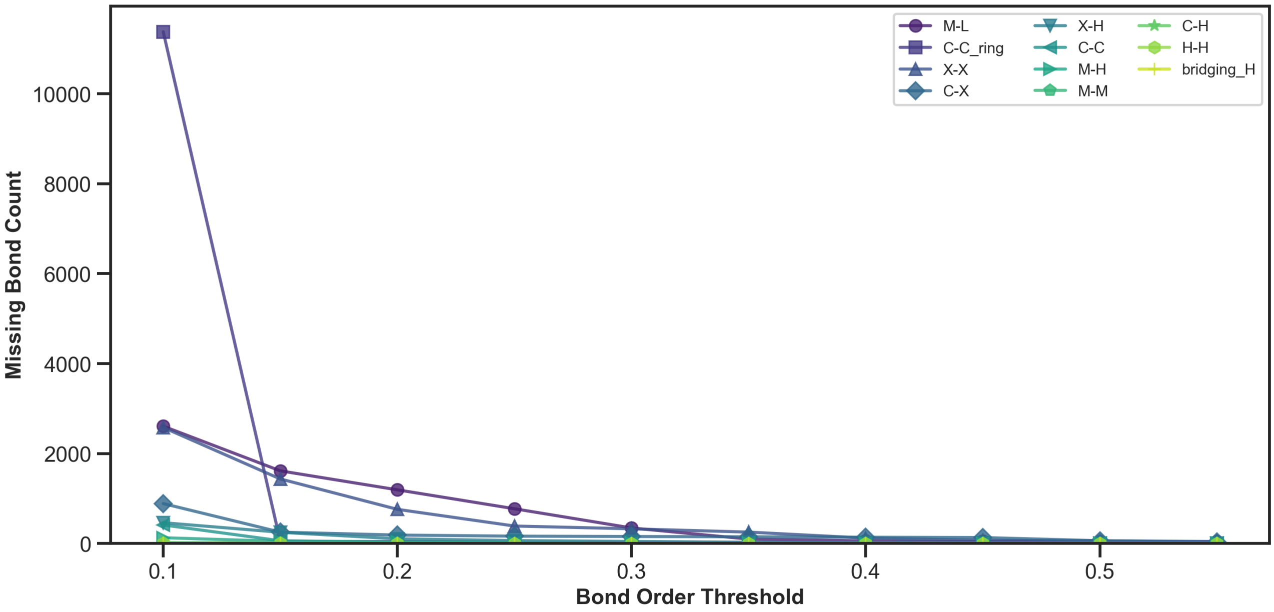

Figure S4: Total missing count of bonds compared to DFT as ground truth across different BO threshold values.

1.4 Element Performance

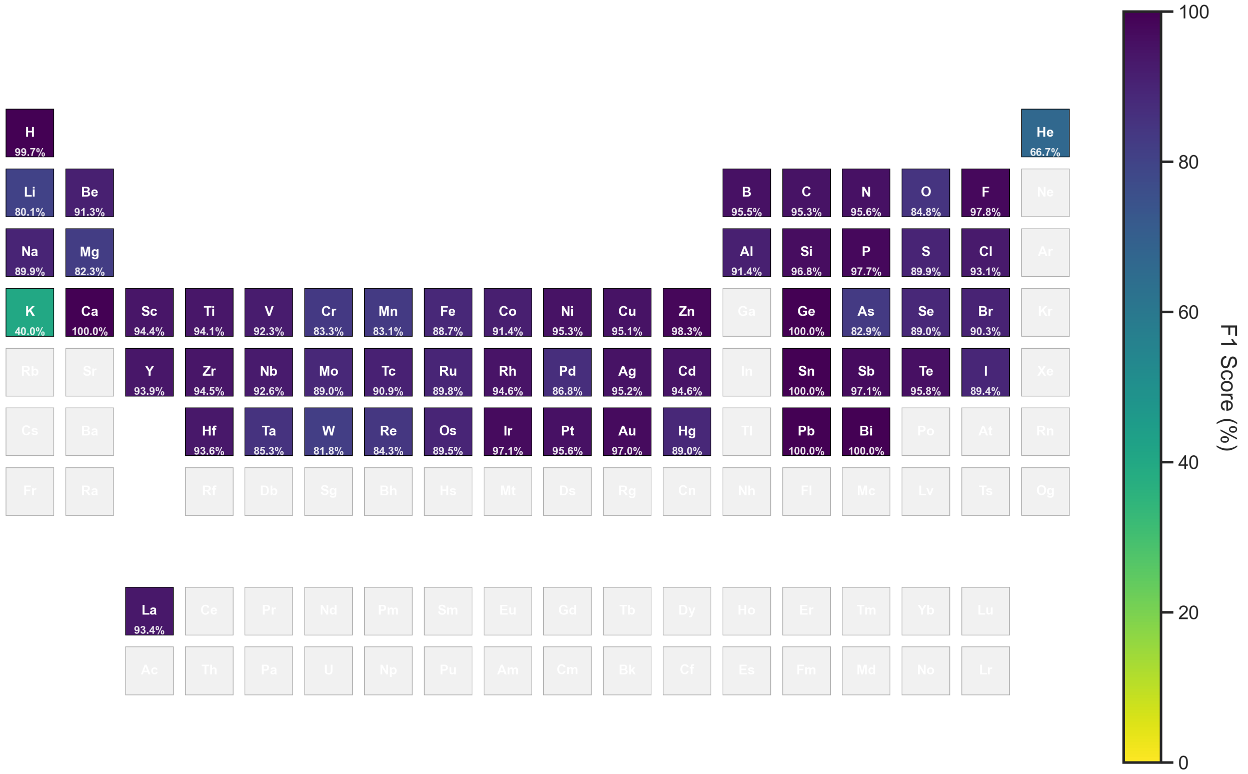

Figure S5: Bond detection performance across the periodic table.

1.5 Random Structures from tmQM

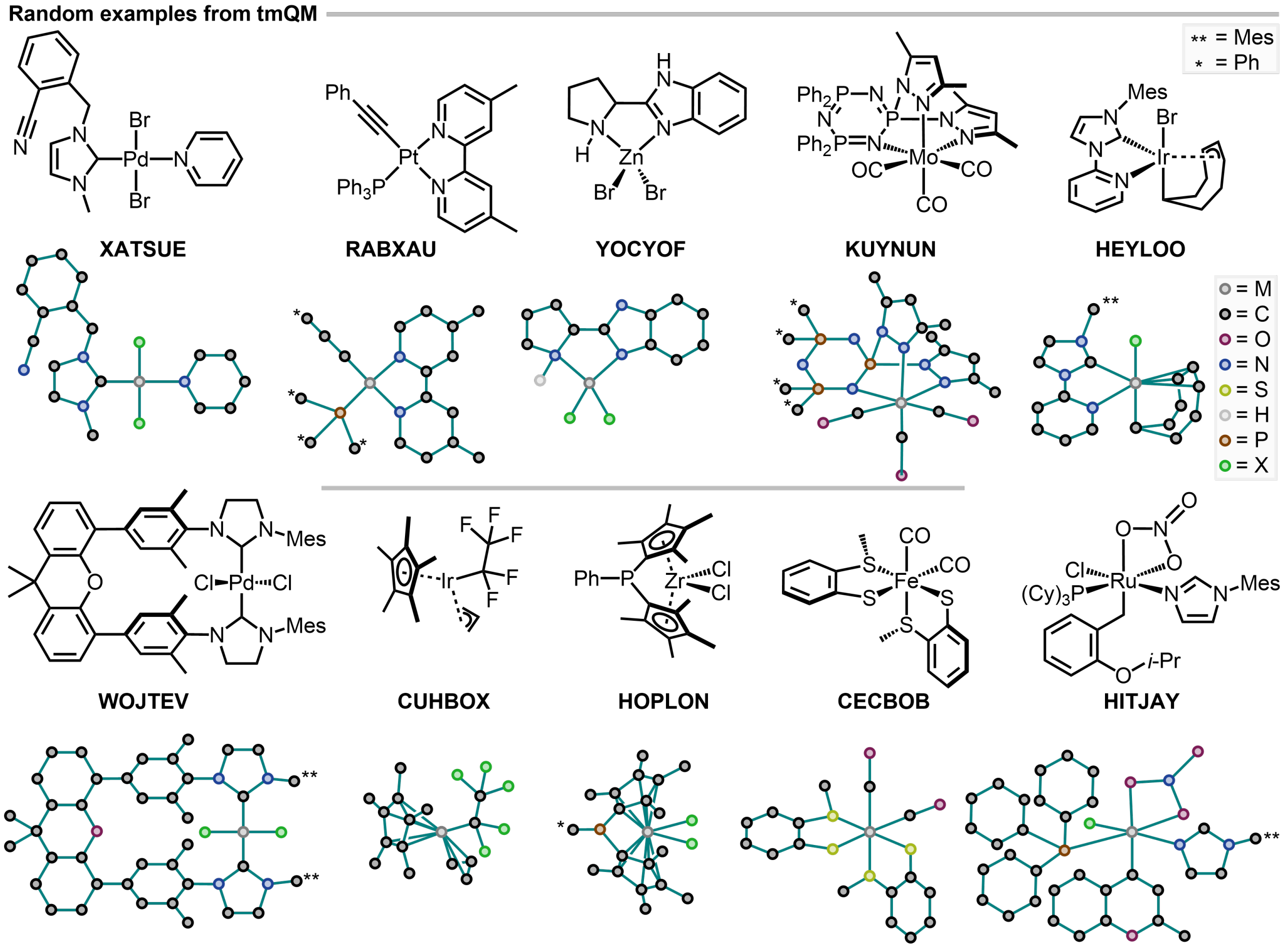

Figure S6: 10 random complexes from tmQM showing 100% accurate molecular connectivity.

2 Output Examples

2.1 S_N2 TS CLI output

```
> graphrc examples/data/sn2_large.out

================================================================================
                                GRAPHRC
            Internal Coordinate Analysis of Vibrational Modes
                          A. S. Goodfellow, 2025
================================================================================

Version:        graphRC v1.3.5
Dependency:     xyzgraph v1.4.8
Citations:   1) A. S. Goodfellow, graphRC: Internal Coordinate Analysis of
                Vibrational Modes, v1.3.5, 2025,
                https://github.com/aligfellow/graphRC.git.
             2) A. S. Goodfellow, xyzgraph: Molecular Graph Construction from
                Cartesian Coordinates, v1.4.8, 2025,
                https://github.com/aligfellow/xyzgraph.git.
Input:          sn2_large.out

Reading trajectory from sn2_large.out
Loaded 20 frames from trajectory
Using TS frame: 0
Selected diverse frames for analysis: [5, 14]

Analyzed Mode 0: -606.23 cm¹ (imaginary)

First 5 non-zero vibrational frequencies:
  Mode 0: -606.23 cm¹ (imaginary)
  Mode 1: 7.64 cm¹
  Mode 2: 16.09 cm¹
  Mode 3: 36.26 cm¹
  Mode 4: 46.70 cm¹

================================================================================
                    VIBRATIONAL TRAJECTORY ANALYSIS
================================================================================

=========================== Significant Bond Changes ===========================

Bond (0, 21)  [C-N]  Δ =   1.666 Å,  Initial =   2.158 Å
Bond (0, 1)   [C-I]  Δ =   1.310 Å,  Initial =   2.563 Å

================================================================================
```

2.2 N-inversion TS CLI output

```
> graphrc examples/data/inversion.v000.xyz -g
================================================================================
                                GRAPHRC
            Internal Coordinate Analysis of Vibrational Modes
                          A. S. Goodfellow, 2025
================================================================================

Version:        graphRC v1.3.5
Dependency:     xyzgraph v1.4.8
Citations:   1) A. S. Goodfellow, graphRC: Internal Coordinate Analysis of
                Vibrational Modes, v1.3.5, 2025,
                https://github.com/aligfellow/graphRC.git.
             2) A. S. Goodfellow, xyzgraph: Molecular Graph Construction from
                Cartesian Coordinates, v1.4.8, 2025,
                https://github.com/aligfellow/xyzgraph.git.
Input:          inversion.v000.xyz
Parameters:     enable_graph=True

Reading trajectory from inversion.v000.xyz
Loaded 20 frames from trajectory
Using TS frame: 0
Selected diverse frames for analysis: [5, 14]
Characterizing vibrational mode...
Running graph-based analysis...
Graph building mode: TS-centric (with guided bonding)
...
...

================================================================================
                         MODE CHARACTERIZATION
================================================================================

Mode Type: INVERSION
Description: N inversion with methyl group motion

Inversion at atom 1 (N)
  100% of dihedrals involve this atom
  Moving group: methyl group
  Max displacement: 0.177 Å

================================================================================
                    VIBRATIONAL TRAJECTORY ANALYSIS
================================================================================

========================= Significant Dihedral Changes =========================

Dihedral (0, 1, 7, 20)  [C-N-C-H]  Δ =  62.713 °,  Initial =   0.033 °
Dihedral (6, 0, 1, 7)   [C-C-N-C]  Δ =  48.581 °,  Initial = 124.372 °
Dihedral (7, 1, 2, 3)   [C-N-C-C]  Δ =  47.590 °,  Initial = 124.337 °

================================================================================
```

2.3 BIMP TS CLI output

```
> graphrc examples/data/bimp.out
```

```
================================================================================
                                GRAPHRC
            Internal Coordinate Analysis of Vibrational Modes
                          A. S. Goodfellow, 2025
================================================================================

Version:        graphRC v1.3.5
Dependency:     xyzgraph v1.4.8
Citations:   1) A. S. Goodfellow, graphRC: Internal Coordinate Analysis of
                Vibrational Modes, v1.3.5, 2025,
                https://github.com/aligfellow/graphRC.git.
             2) A. S. Goodfellow, xyzgraph: Molecular Graph Construction from
                Cartesian Coordinates, v1.4.8, 2025,
                https://github.com/aligfellow/xyzgraph.git.
Input:          bimp.out

Reading trajectory from bimp.out
Loaded 20 frames from trajectory
Using TS frame: 0
Selected diverse frames for analysis: [5, 15]

Analyzed Mode 0: -333.88 cm¹ (imaginary)

First 5 non-zero vibrational frequencies:
  Mode 0: -333.88 cm¹ (imaginary)
  Mode 1: 8.57 cm¹
  Mode 2: 12.72 cm¹
  Mode 3: 13.27 cm¹
  Mode 4: 15.83 cm¹

================================================================================
                    VIBRATIONAL TRAJECTORY ANALYSIS
================================================================================

=========================== Significant Bond Changes ===========================

Bond (11, 12)  [O-C]  Δ =   2.052 Å,  Initial =   2.064 Å
Bond (10, 14)  [C-C]  Δ =   0.426 Å,  Initial =   2.656 Å

================================================================================
```

3 Transition State Examples

3.1 IRC verified

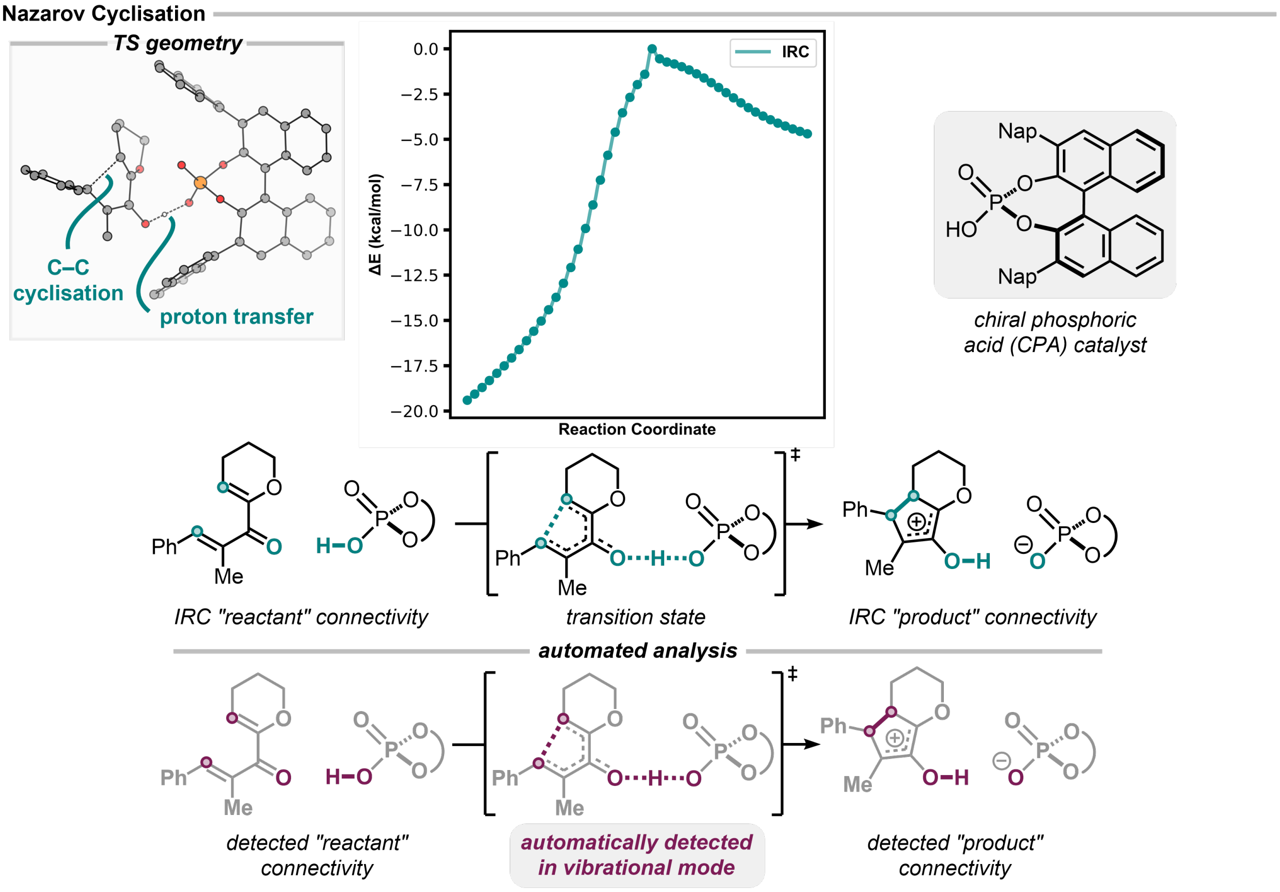

Figure S7: Nazarov cyclisation example.[3]

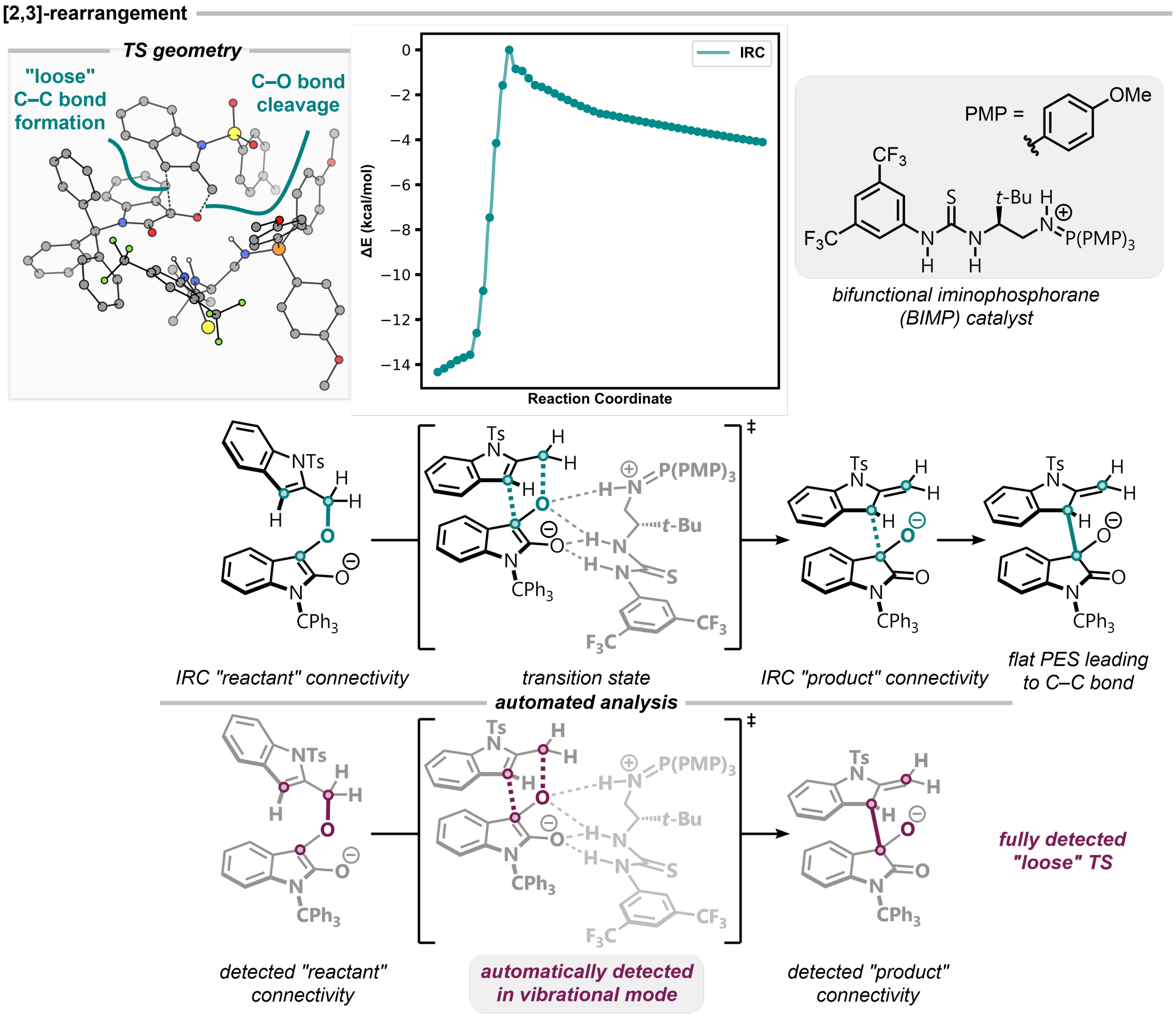

Figure S8: BIMP catalysed 2, 3]-rearrangement example.[4]

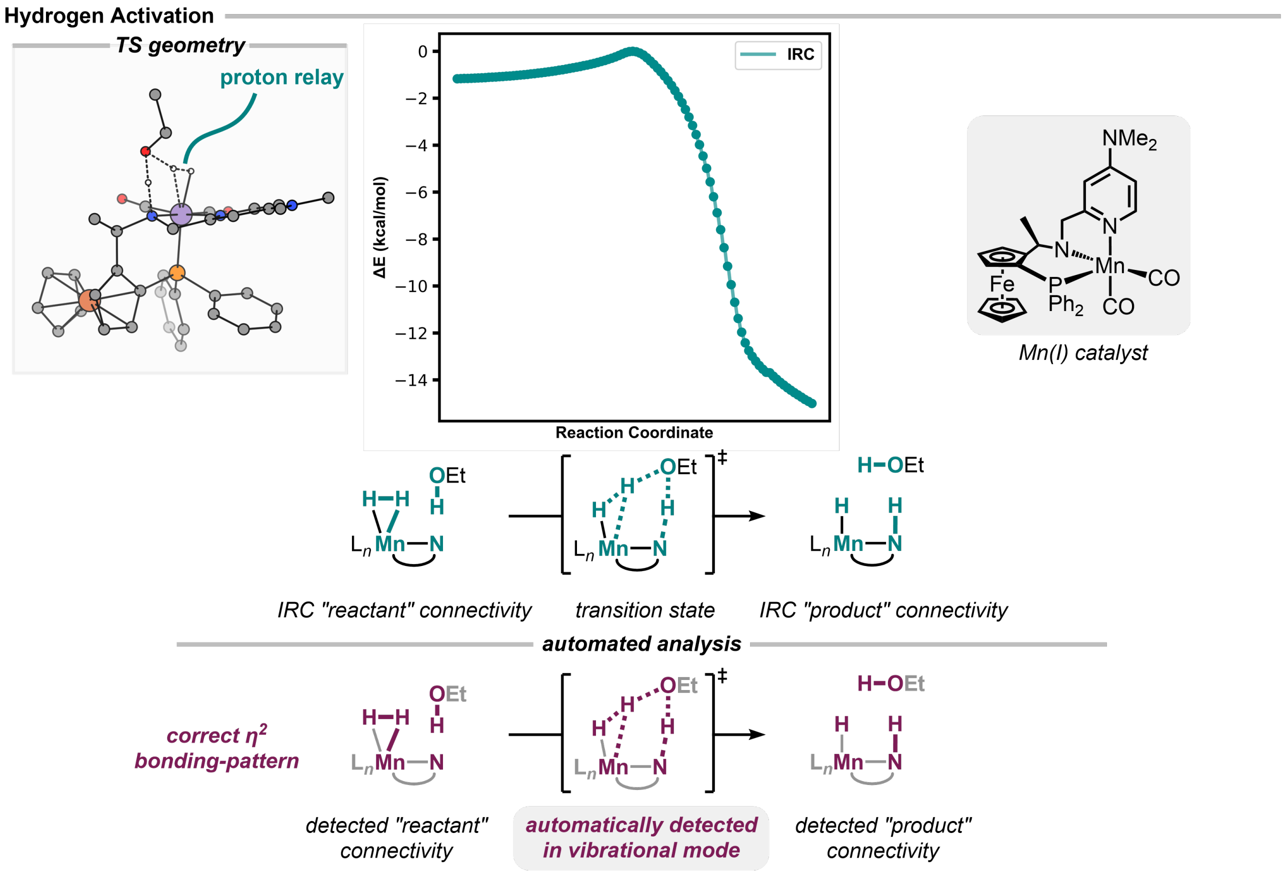

Figure S9: Manganese hydrogen activation example.[5]

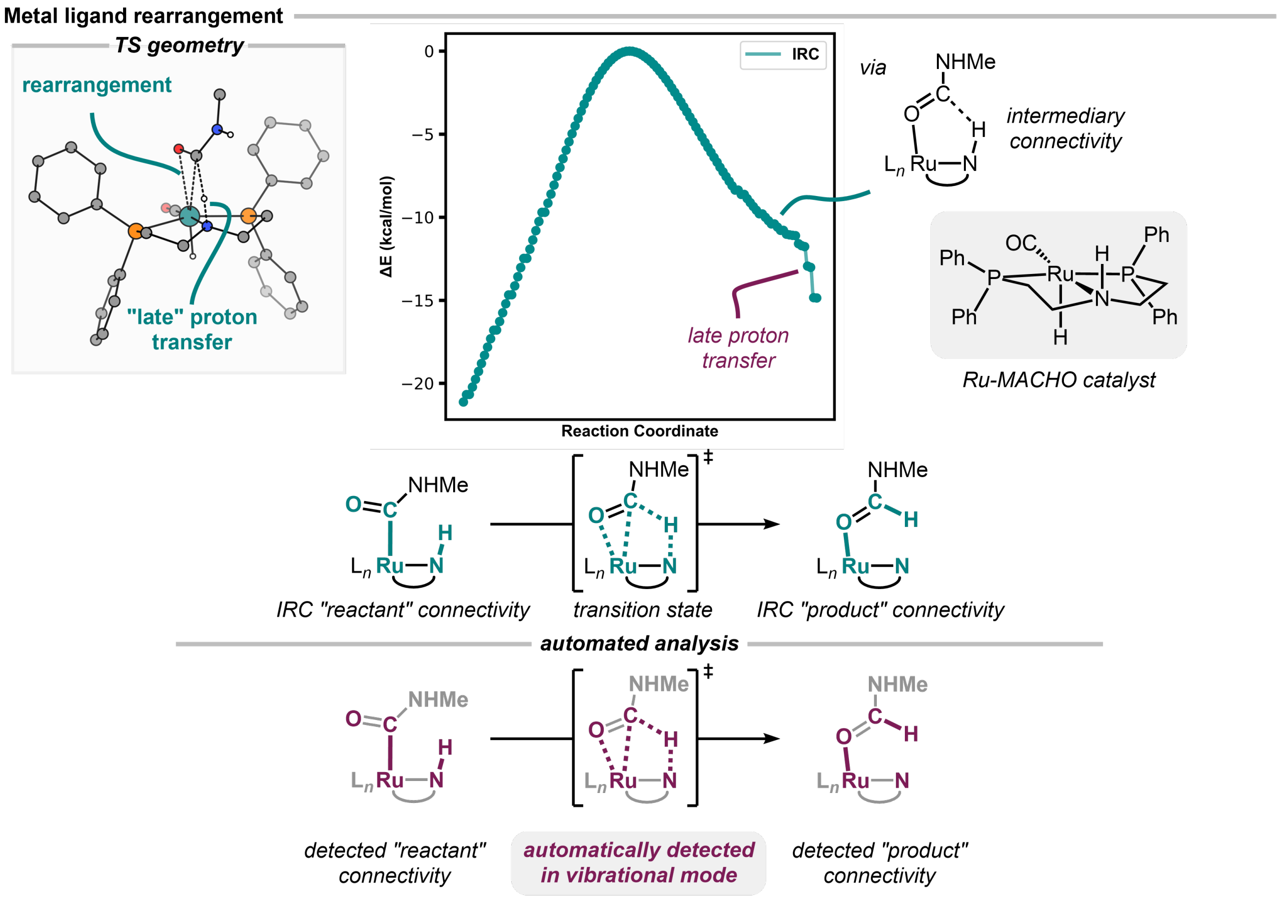

Figure S10: Ruthenium ligand rearrangement example.[6]

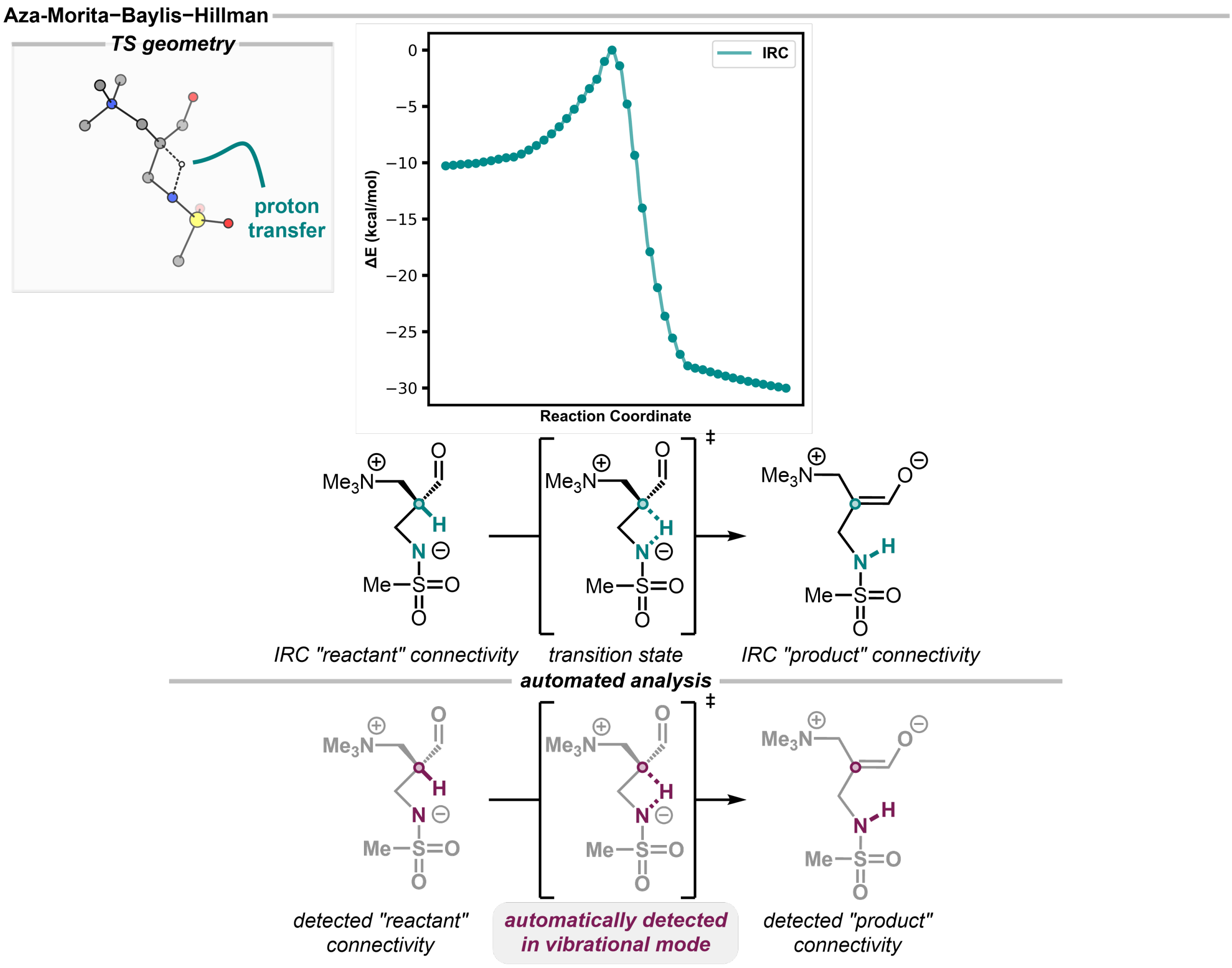

Figure S11: Aza-Morita-Baylis-Hillman example.[7]

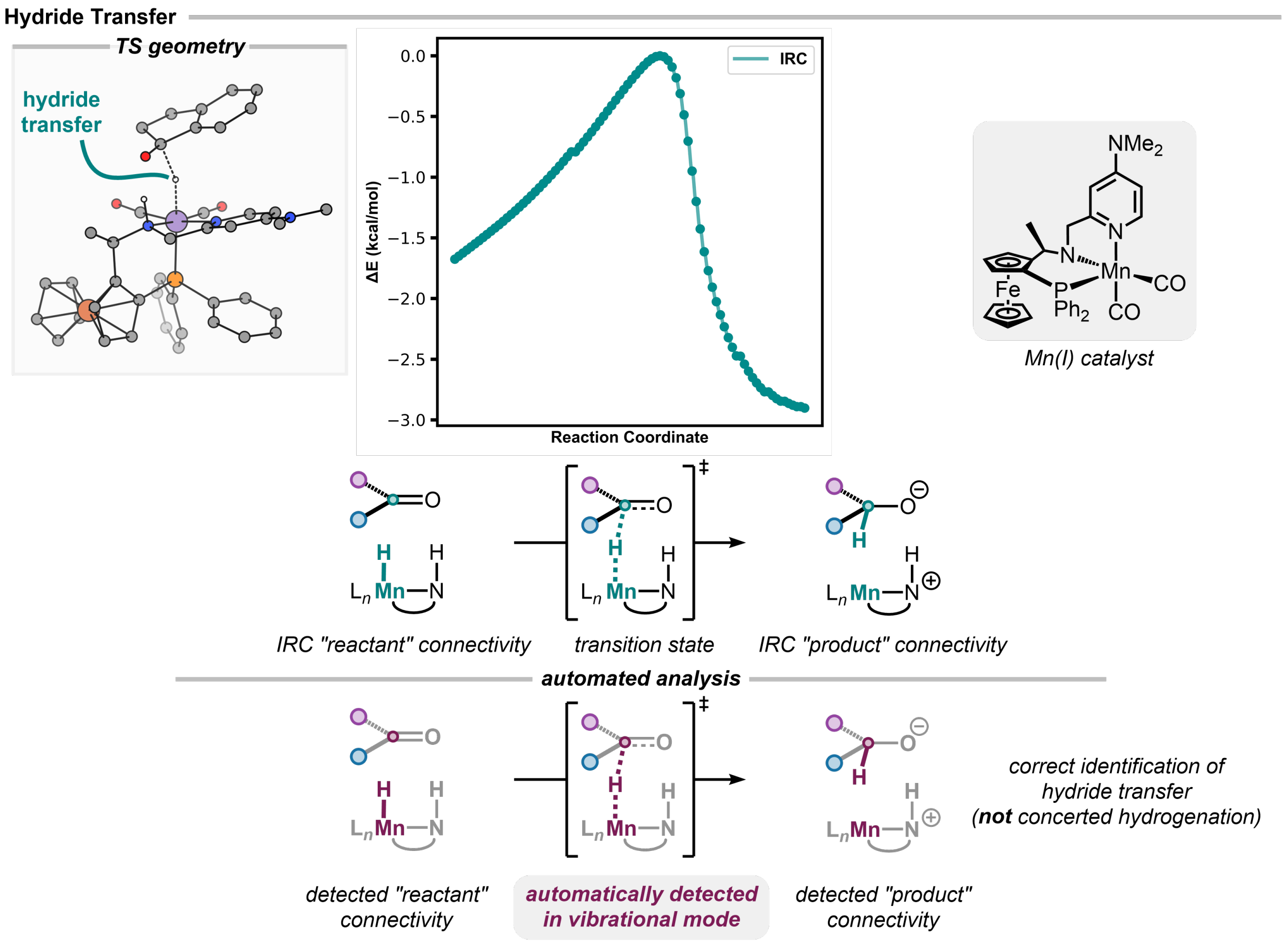

Figure S12: Manganese hydride transfer example.[5]

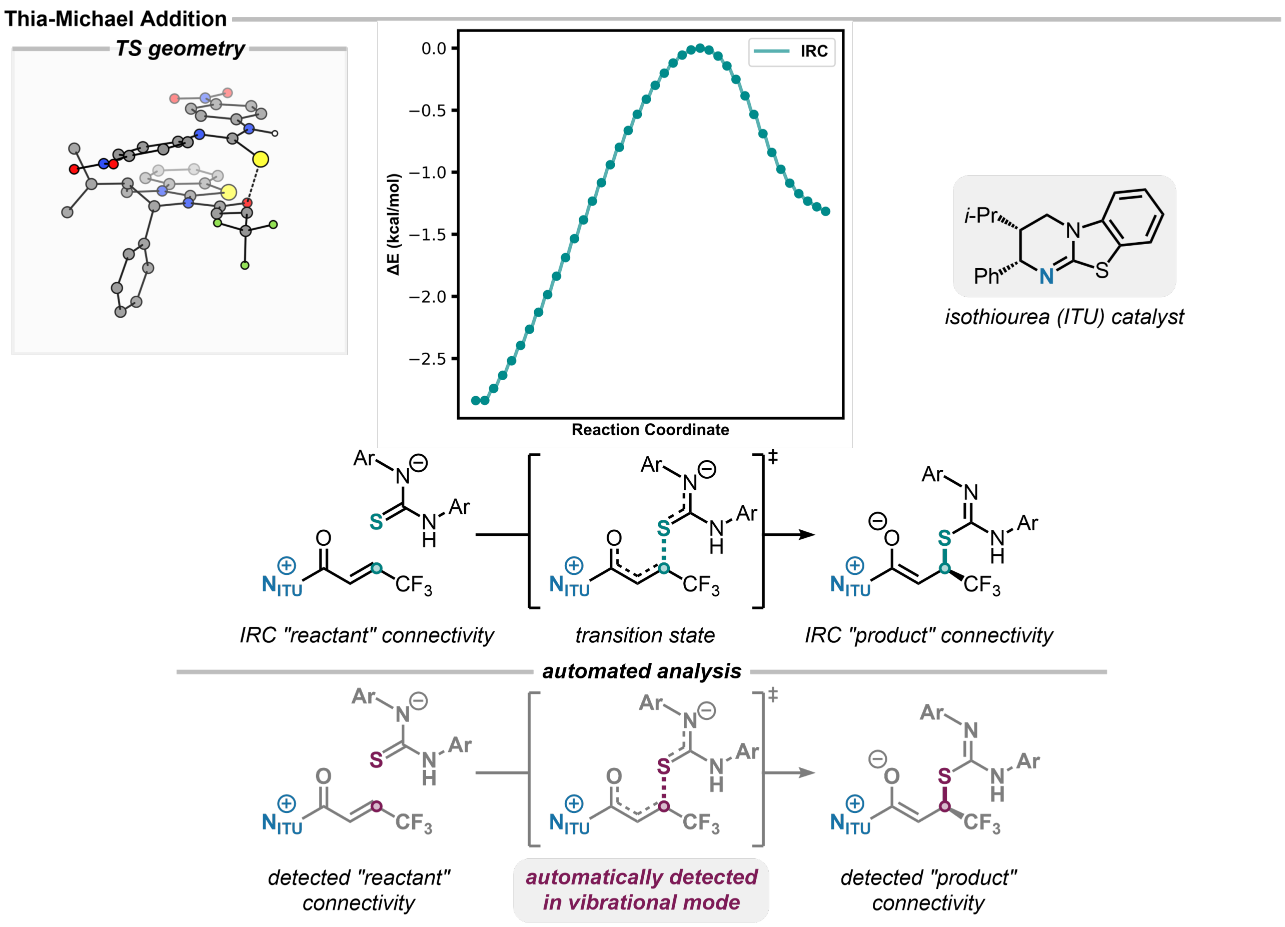

Figure S13: Thia-Michael addition example.[8]

3.2 QRC verified

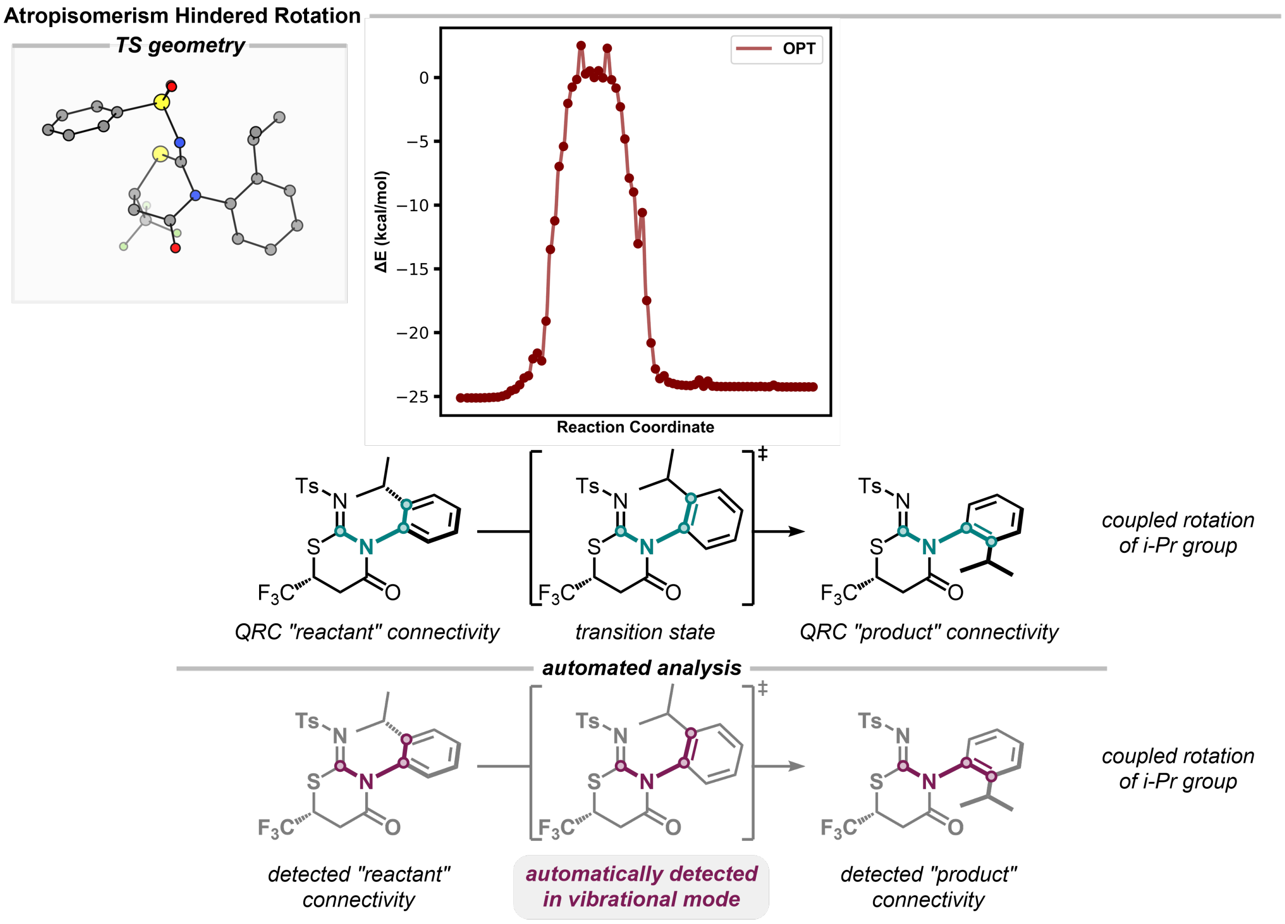

Figure S14: Atropisomeric hindered rotation example.[8] Profile obtained from optimisation of the transition state displaced along the normal mode.

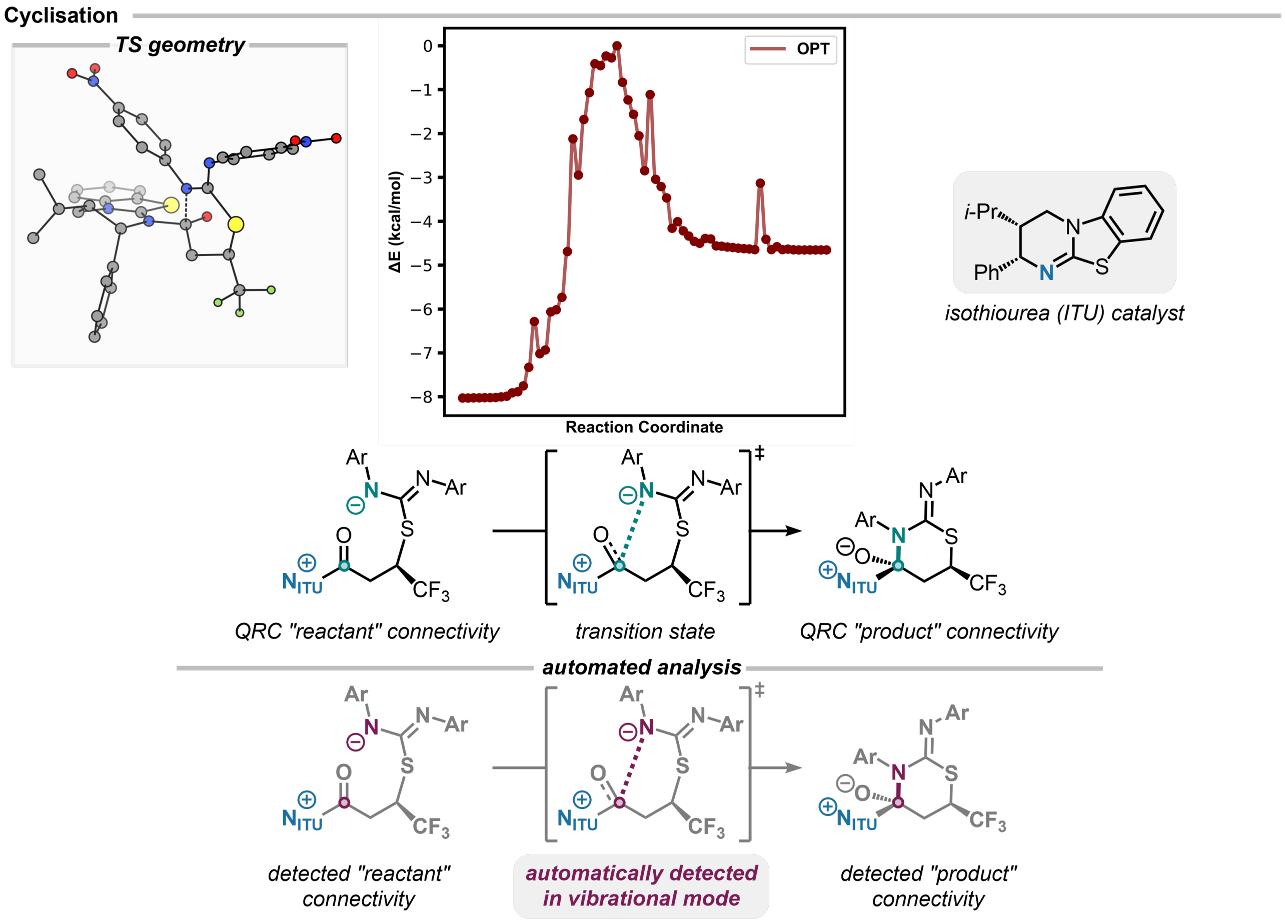

Figure S15: Cyclisation example.[8] Profile obtained from optimisation of the transition state displaced along the normal mode.

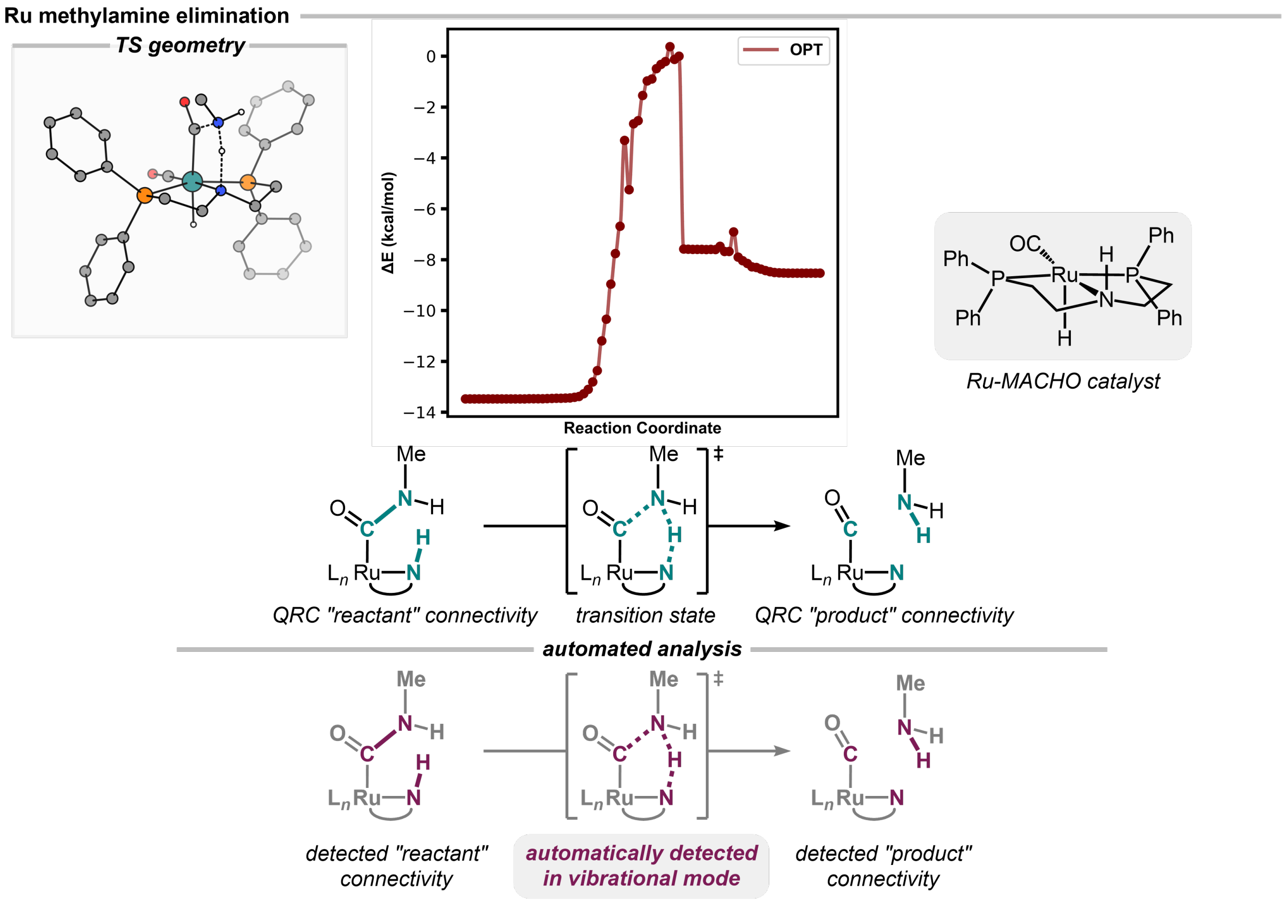

Figure S16: Ruthenium elimination of methylamine example.[6] Profile obtained from optimisation of the transition state displaced along the normal mode.

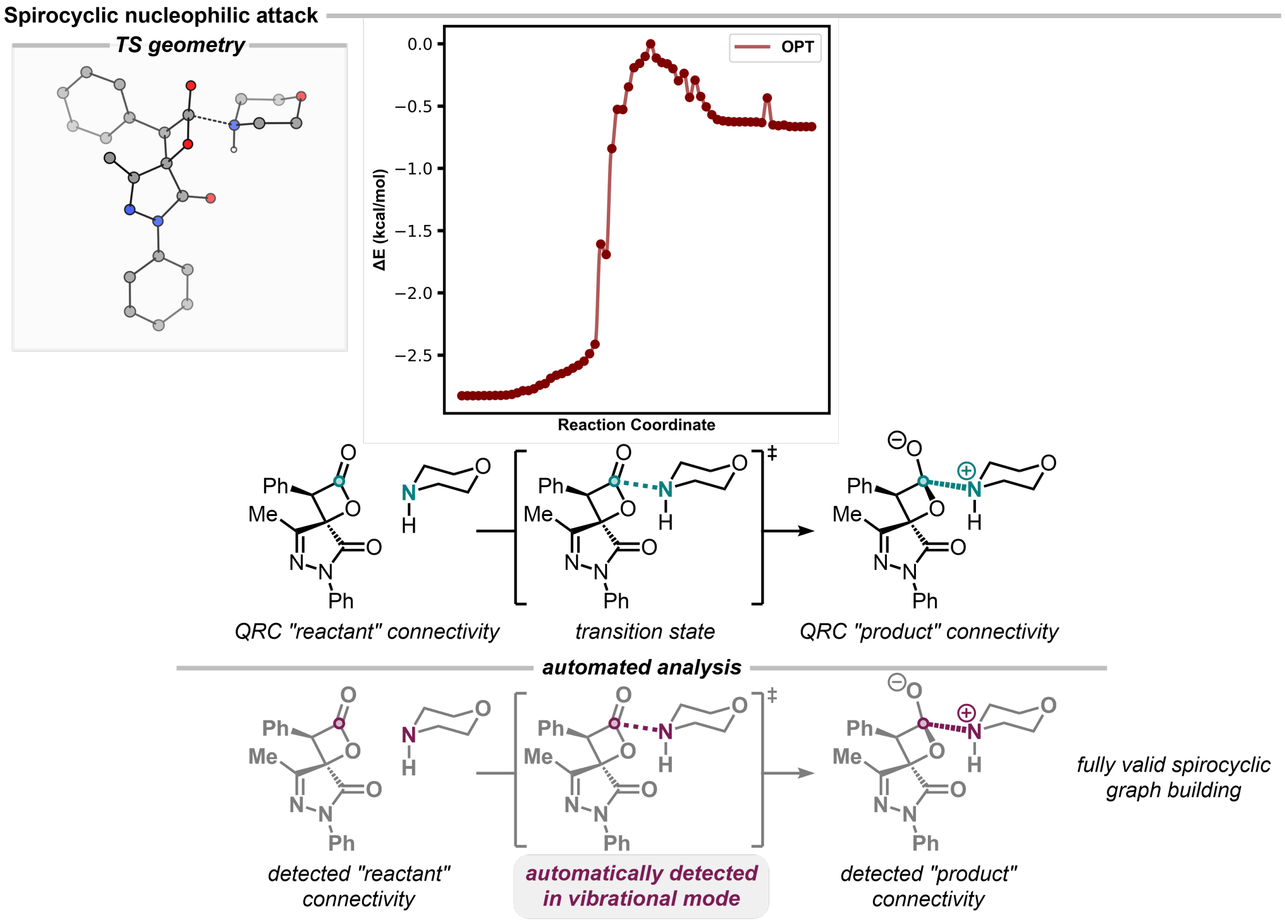

Figure S17: Spirocyclic nucleophilic attack example.[9] Profile obtained from optimisation of the transition state displaced along the normal mode.

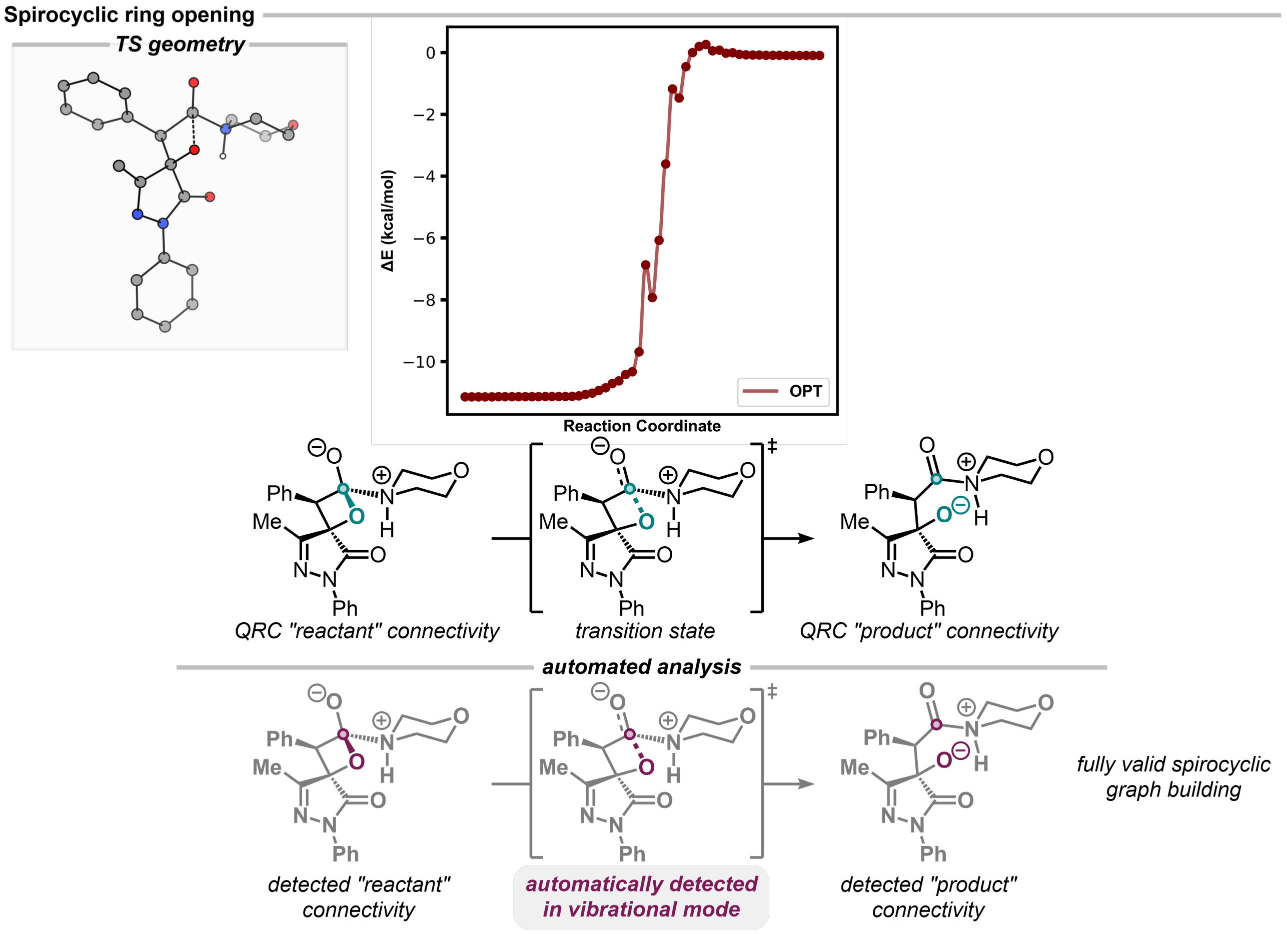

Figure S18: Spirocyclic ring opening example.[9] Profile obtained from optimisation of the transition state displaced along the normal mode.

4 High Throughout Validation

To validate the approach, we obtain a diverse transitions state database from reference [10], which provides GFN2-xTB[11] optimised TS geometries, alongside corresponding IRC trajectories computed using using YARP.[12] For tractability, we perform a random sample of the dataset, obtaining transition state geometries and IRC trajectories for these. In a similar method to the publication from Savoie *et al.*, we performed single-point calculations across all charge-multiplicity combinations (charges = −2, −1, 0, +1, +2; multiplicities = 1, 2, 3). We evaluate the charge-multiplicity combination with the lowest gradient at this geometry, which corresponds to the charge-multiplicity combination used for evaluation of the stationary point in the dataset. A GFN2-xTB Hessian was then evaluated on this geometry to analyse the imaginary mode; however, inconsistencies between computational packages frequently led to the identification of multiple imaginary modes. To eliminate this numerical noise, each TS was reoptimised using a short GFN2-xTB NEB-TS calculation in ORCA.[13] The NEB calculations were restricted to 50 steps to ensure the optimisation recovered the original TS, avoiding convergence to an alternative TS. Calculations that were not fully converged and contained more than one imaginary modes were discarded. QRC calculations were performed using GFN2-xTB with 'extreme' optimisation criteria. To ensure that the TS located corresponded to the original IRC path, the connectivity of the reactant and product were compared between the QRC and IRC trajectories, leaving 395 TS for analysis.

4.1 Stratified Performance

Table S2: Performance of the vibrational mode analysis stratified by number of bond changes.

Number of Bond Changes	N samples	True Positives	False Positives	F1 Score
1	79	100.0%	16.0%	91.3%
2	177	91.8%	3.0%	94.3%
3	69	72.9%	3.2%	83.2%
4	49	74.5%	1.4%	84.9%
5	13	75.4%	0.0%	86.0%
6	5	53.3%	0.0%	69.6%
7+	3	61.9%	0.0%	76.5%

4.2 Random Examples

As a random sample of the dataset was taken from reference [10], the transition states will statistically follow the distribution of the original dataset. These contain diverse chemical bonding across the whole periodic table, for further information see reference [10]. To illustrate the breadth and complexity of the transition states analysed in this work, we present a selection of 20 random examples in **Figures** S19–S22 with a breakdown of the bond changes that were detected, missed and falsely detected with the normal mode analysis. In 100% of examples, the key bond changes were identified, showing the robustness of approach across transition states of varied complexity.

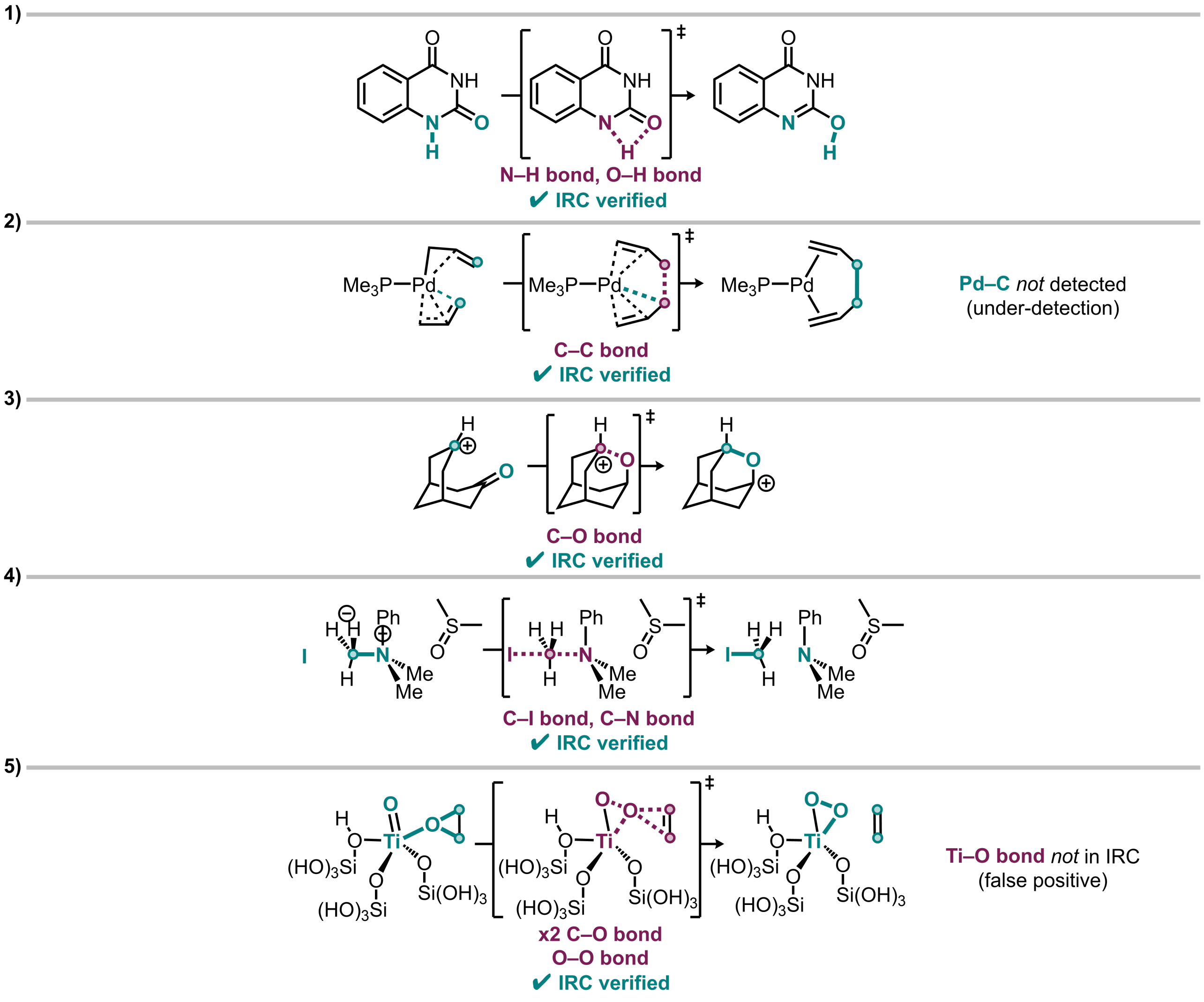

Figure S19: **1/4** – 20 random examples of transition states analysed in this work from the dataset accompanying reference [10].

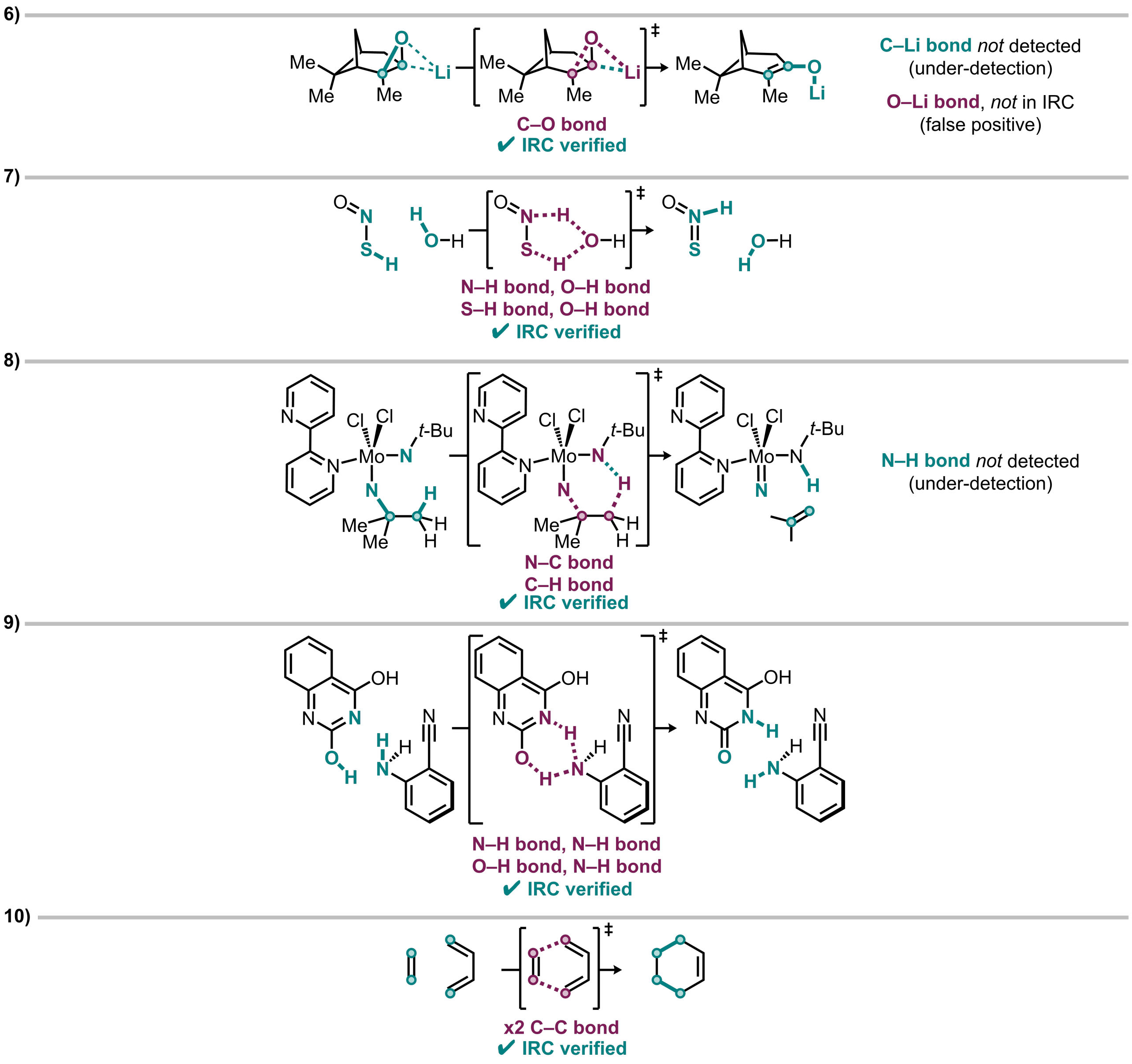

Figure S20: **2/4** – 20 random examples of transition states analysed in this work from the dataset accompanying reference [10].

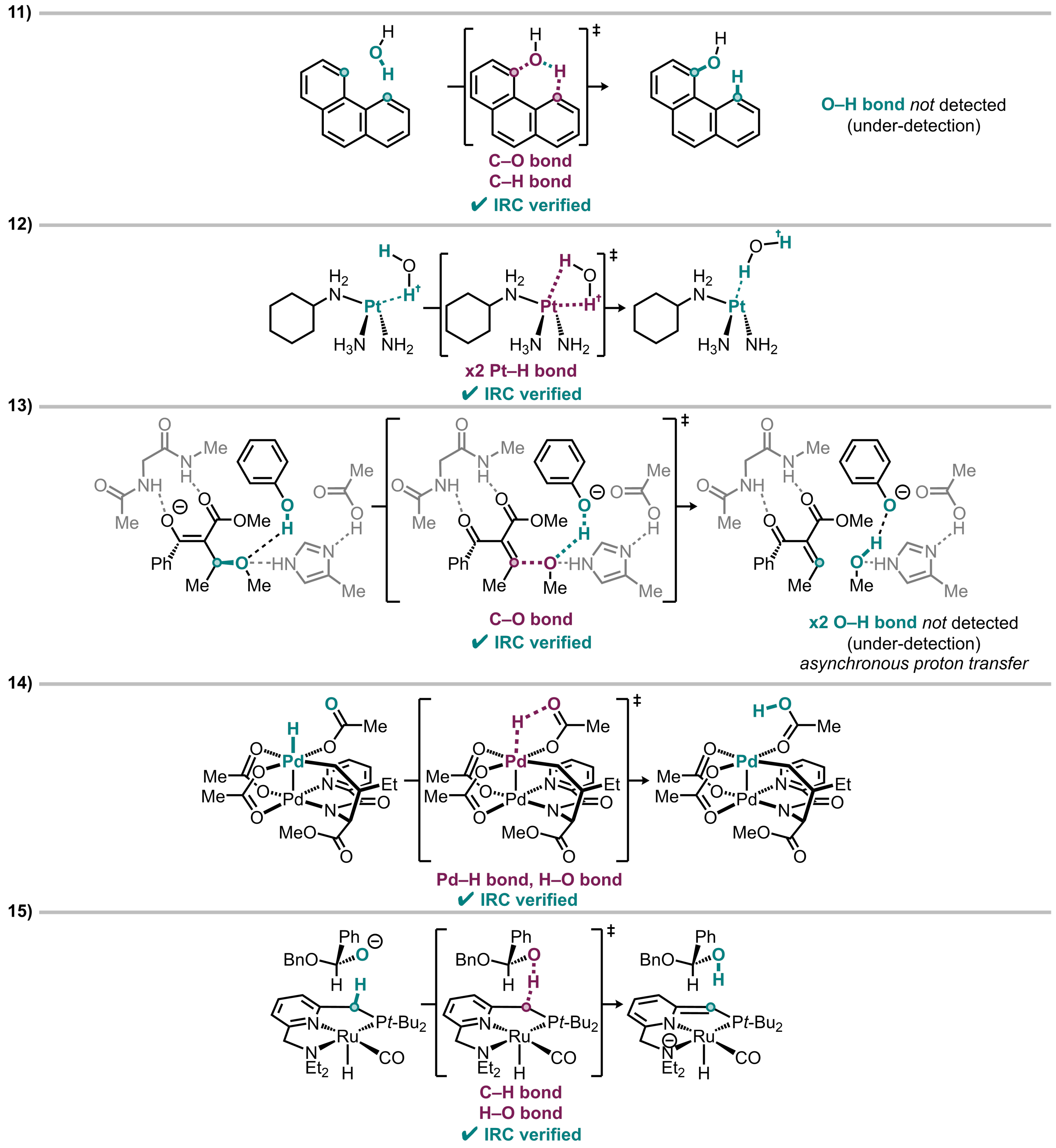

Figure S21: **3/4** – 20 random examples of transition states analysed in this work from the dataset accompanying reference [10].

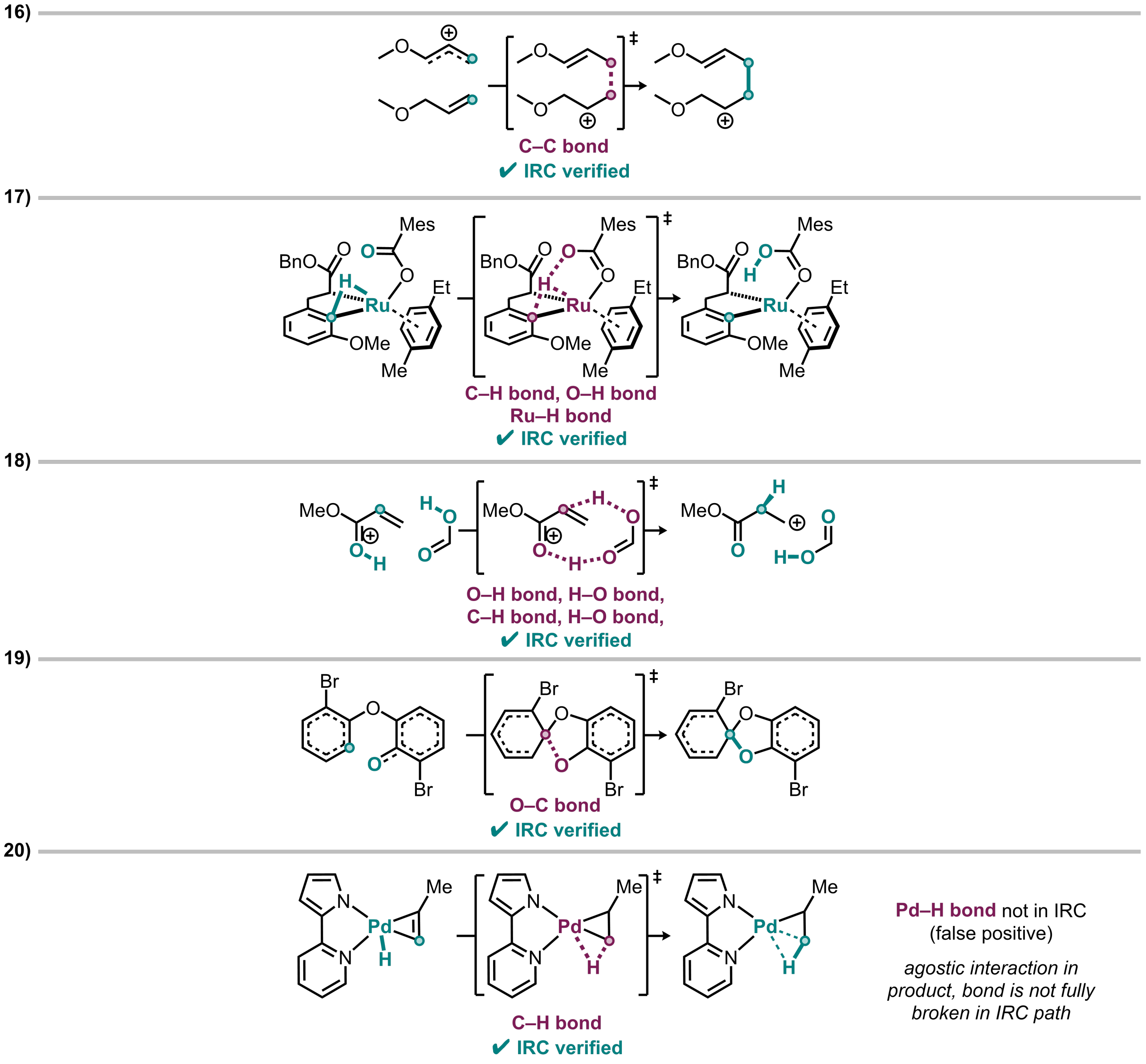

Figure S22: **4/4** – 20 random examples of transition states analysed in this work from the dataset accompanying reference [10].

4.3 Low Accuracy High-Throughput Examples

The high-throughput TS with an F1 score <50% relative to the IRC path are shown in **Figures S23–S24**. As outlined below, these highly concerted examples are a consequence of both the GFN2-xTB level of theory and some partial TS extraction. They remain valid TS at the GFN2-xTB level of theory but represent a slightly different PES compared to DFT.

1. Reference [14]. Incomplete structure of **Tautomer 1-2w-Ph**. This is likely due to the column splitting of the Cartesian coordinates in the SI `.pdf` file during extraction with [10]. The TS is valid at the GFN2-xTB level of theory; however, this is not reflective of realistic chemical reactivity.

2. Reference [15]. **3aTS**. GFN2-xTB has resolved an overly concerted mechanism compared to the DFT calculations in the original manuscript (IRC validated at PBE0/TZVP).

3. Reference [16]. **E'TS2**. GFN2-xTB has led to a ligand dissociation which is not present in the DFT optimised TS ($M06_{SMD(DCM)}$/6-311G(d,p)/def2-TZVP(Ru)//$M06_{SMD(DCM)}$/6-31G(d)/SDD(Ru)).

4. Reference [17]. Incomplete structure of a C_{60} structure. This is likely due to the column splitting of the Cartesian coordinates in the SI `.pdf` file during extraction with [10]. This is a valid TS at the GFN2-xTB level of theory; however, this complex TS is not reflective of realistic chemical reactivity.

5. Reference [18]. Incomplete structure of a TS from the manuscript. This is likely due to the column splitting of the Cartesian coordinates in the SI `.pdf` file during extraction with [10]. This is a valid TS at the GFN2-xTB level of theory; however, this highly concerted TS is not reflective of realistic chemical reactivity.

6. Reference [19]. **TS N-Cl 7w** (with $(CH_3)_2NH$ and HOCl). This GFN2-xTB TS is consistent with the DFT TS (B3LYP/6-31G(d)), with multiple water molecules acting as an extended protic relay.

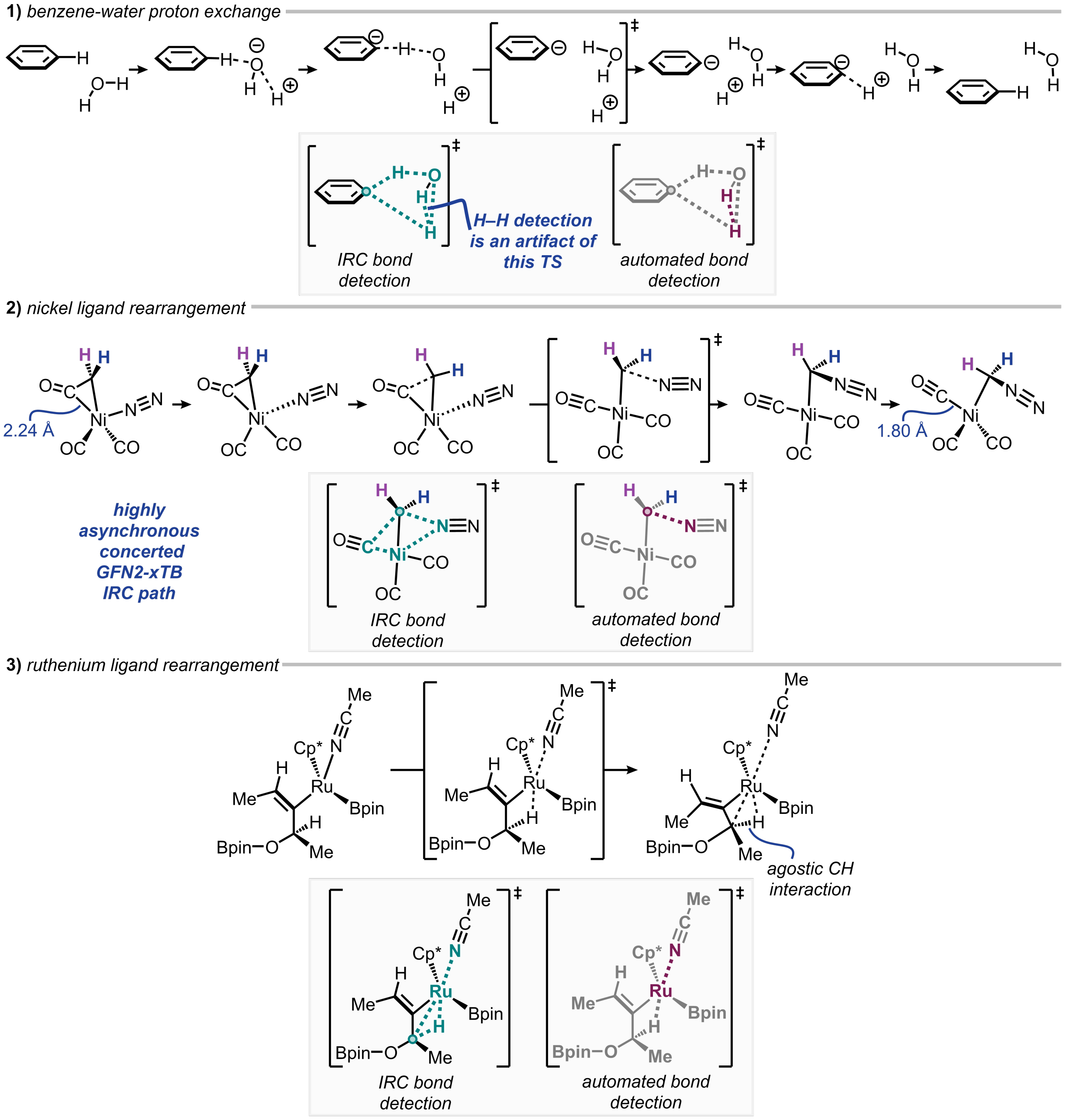

Figure S23: **1/2** – Poor performing examples with an F1 score <50%.

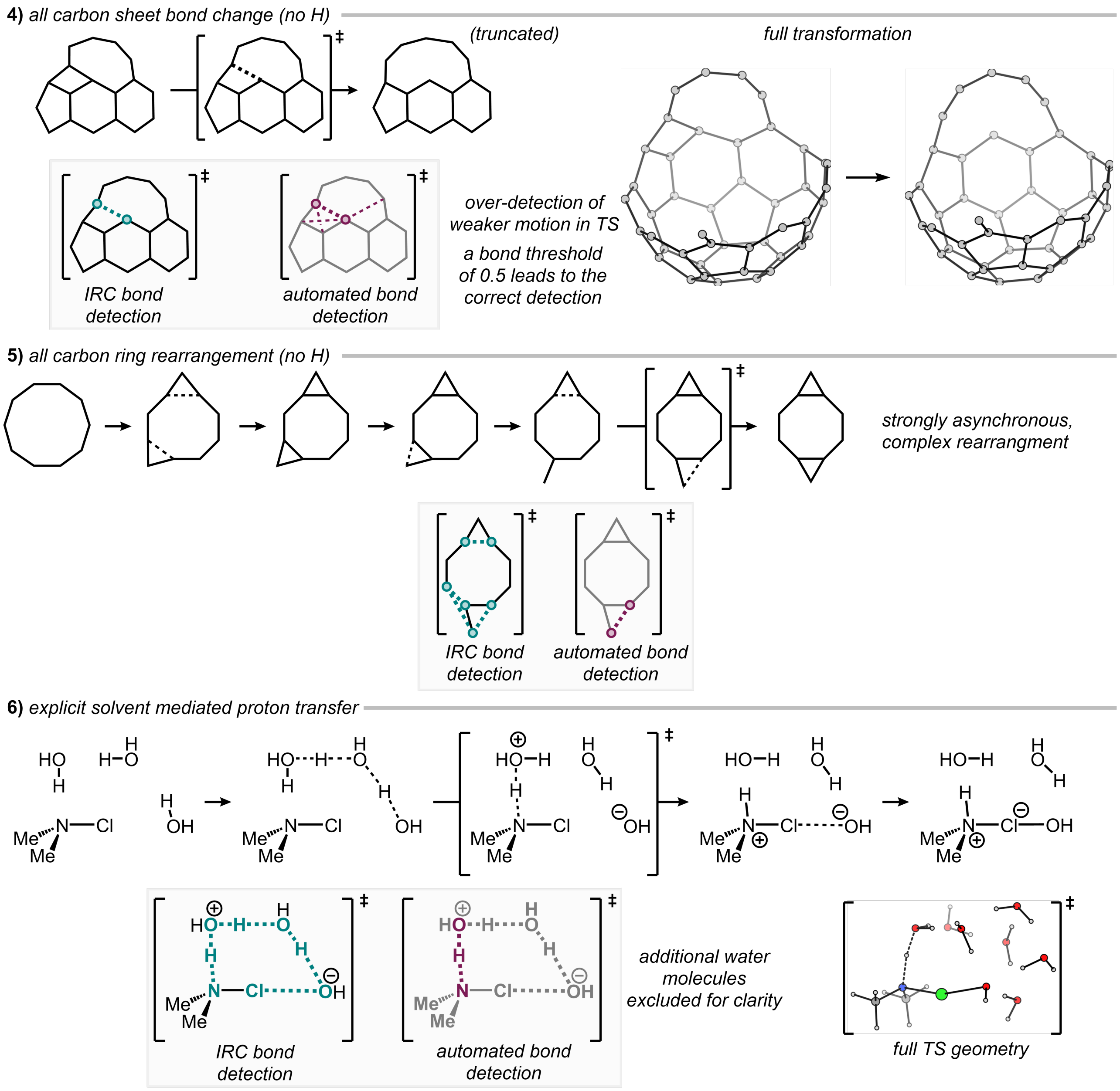

Figure S24: **2/2** – Poor performing examples with an F1 score <50%.

4.4 Relaxed TS Criteria

With less stringent TS validation criteria, we compare the performance of 1995 TS with a single imaginary mode, without enforcing that the QRC connectivity is consistent with the IRC connectivity. This dataset expands coverage across the periodic table, particularly for transition-metal and heavy p-block TS. This does so with the introduction of more uncertainty in the ground truth connectivity of the TS. A lower F1 score of 84.5% is obtained across 5346 'ground truth' bonds. The element pair performance is shown in **Figure** S25.

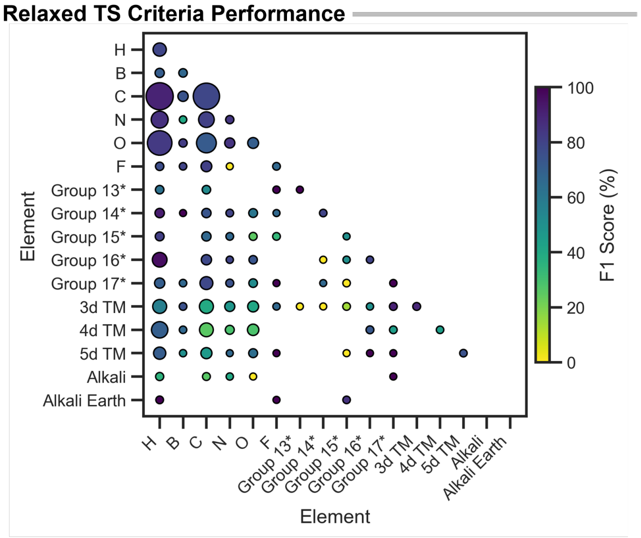

Figure S25: Extended element pair performance (F1 score %) across 1995 examples relative to GFN2-xTB derived IRC connectivity. Heavy p-group elements are grouped for clarity. An asterisk (*) denotes that these groupings exclude first row p-block elements (<Ne) which are shown individually. Marker size is proportional to the frequency of the element-pair, coloured by F1 score.

References

(1) L. Goerigk, A. Hansen, C. Bauer, S. Ehrlich, A. Najibi and S. Grimme, *Phys. Chem. Chem. Phys.*, 2017, **19**, 32184–32215.

(2) D. Balcells and B. B. Skjelstad, *J. Chem. Inf. Model.*, 2020, **60**, 6135–6146.

(3) Y. P. Chin and E. H. Krenske, *J. Org. Chem.*, 2021, **87**, 1710–1722.

(4) T. Kang, J. O'Yang, K. Kasten, S. S. Allsop, T. Lewis-Atwell, E. H. Farrar, M. Juhl, D. B. Cordes, A. P. McKay, M. N. Grayson and A. D. Smith, *Nature Chemistry 2026*, 2026, 1–10.

(5) A. S. Goodfellow, M. L. Clarke and M. Bühl, *Chem. Eur. J.*, 2025, **31**, e202501063.

(6) J. Luk, A. S. Goodfellow, N. D. More, M. Bühl and A. Kumar, *Chem. Sci.*, 2024, **15**, 16594–16604.

(7) D. Roy, C. Patel and R. B. Sunoj, *J. Org. Chem*, 2009, **74**, 6936–6943.

(8) A. J. Nimmo, A. S. Goodfellow, J. T. Guntley, A. P. Mckay, D. B. Cordes, M. Bühl and A. D. Smith, *Chem. Sci.*, 2025, **16**, 10494.

(9) A. Conboy, A. S. Goodfellow, K. Kasten, J. Dunne, D. B. Cordes, M. Bühl and A. D. Smith, *Chem. Sci.*, 2024, 52–69.

(10) Z. Li, I. Y. Yang and B. M. Savoie, *ChemRxiv*, 2025, DOI: `10.26434/chemrxiv-2025-ccgfs`.

(11) C. Bannwarth, S. Ehlert and S. Grimme, *J. Chem. Theory Comput,* 2019, **15**, 1652–1671.

(12) Q. Zhao and B. M. Savoie, *Nat. Comput. Sci.*, 2021, **1**, 479–490.

(13) F. Neese, *WIREs Comput. Mol. Sci.*, 2025, **15**, e70019.

(14) S. Mirzaei, A. A. Taherpour and H. Khalilian, *ChemistrySelect*, 2018, **3**, 6042–6049.

(15) B. Barcs, L. László, L. Lászlókollár, T. Tamás, K. Kégl and J. J. Szentágothai, *Organometallics*, 2012, **31**, 8082–8097.

(16) Q. Feng, S. Li, Z. Li, Q. Yan, X. Lin, L. Song, X. Zhang, Y.-D. Wu and J. Sun, *J. Am. Chem. Soc*, 2022, **144**, 14846–14855.

(17) C. X. Cui, J. R. He, L. B. Qu, C. X. Li, J. L. Peng and F. Maseras, *Chem. Eur. J.*, 2024, **30**, e202402572.

(18) A. Boddeda, M. M. Hossain, M. Saeed Mirzaei, S. V. Lindeman, S. Mirzaei and R. Rathore, *Org. Chem. Front*, 2020, **7**, 3215.

(19) D. Šakić, M. Hanževački, D. M. Smith and V. Vrček, *Org. Biomol. Chem*, 2015, **13**, 11752.
